# Modulation of γ-Secretase Activity by Multiple Enzyme-Substrate Interactions: Implications in Pathogenesis of Alzheimer's Disease

**DOI:** 10.1371/journal.pone.0032293

**Published:** 2012-03-30

**Authors:** Željko M. Svedružić, Katarina Popović, Ivana Smoljan, Vesna Šendula-Jengić

**Affiliations:** 1 Medical Biochemistry, Faculty of Medicine, University of Rijeka, Rab, Croatia; 2 Neurology and Geriatrics, Faculty of Medicine, University of Rijeka, Rab, Croatia; University of South Florida College of Medicine, United States of America

## Abstract

**Background:**

We describe molecular processes that can facilitate pathogenesis of Alzheimer's disease (AD) by analyzing the catalytic cycle of a membrane-imbedded protease γ-secretase, from the initial interaction with its C99 substrate to the final release of toxic Aβ peptides.

**Results:**

The C-terminal AICD fragment is cleaved first in a pre-steady-state burst. The lowest Aβ42/Aβ40 ratio is observed in pre-steady-state when Aβ40 is the dominant product. Aβ42 is produced after Aβ40, and therefore Aβ42 is not a precursor for Aβ40. The longer more hydrophobic Aβ products gradually accumulate with multiple catalytic turnovers as a result of interrupted catalytic cycles. Saturation of γ-secretase with its C99 substrate leads to 30% decrease in Aβ40 with concomitant increase in the longer Aβ products and Aβ42/Aβ40 ratio. To different degree the same changes in Aβ products can be observed with two mutations that lead to an early onset of AD, ΔE9 and G384A. Four different lines of evidence show that γ-secretase can bind and cleave multiple substrate molecules in one catalytic turnover. Consequently depending on its concentration, NotchΔE substrate can activate or inhibit γ-secretase activity on C99 substrate. Multiple C99 molecules bound to γ-secretase can affect processive cleavages of the nascent Aβ catalytic intermediates and facilitate their premature release as the toxic membrane-imbedded Aβ-bundles.

**Conclusions:**

Gradual saturation of γ-secretase with its substrate can be the pathogenic process in different alleged causes of AD. Thus, competitive inhibitors of γ-secretase offer the best chance for a successful therapy, while the noncompetitive inhibitors could even facilitate development of the disease by inducing enzyme saturation at otherwise sub-saturating substrate. Membrane-imbedded Aβ-bundles generated by γ-secretase could be neurotoxic and thus crucial for our understanding of the amyloid hypothesis and AD pathogenesis.

## Introduction

Alzheimer's disease is a slowly progressing neurodegenerative disorder characterized by steadily advancing dementia that is often coupled with insidious onsets of agnosia, aphasia, and apraxia [1]. The current therapy is only symptomatic, and there is no an effective cure or a preventive treatment available [1]. A large body of basic and pharmaceutical research dedicated to tackle the problem of Alzheimer's disease is providing a steadily growing number of potential targets [2], and some very potent drug candidates [3], [4]. Changes in cholesterol metabolism [5], G-protein coupled receptors [6], Aβ clearance [5], [7], [8], mitochondrial dysfunction [9], or changes in APP metabolism [8] are part of a growing list of cellular processes that have been implicated in the pathogenesis. Different alleged causes of Alzheimer's disease have one focal point, a membrane imbedded protease γ-secretase, the key enzyme for production of toxic amyloid-β (Aβ) peptides [10].

Studies of catalytic mechanism of γ-secretase have presented some unique biochemical and biophysical question and experimental challenges [3], [11], [12]. After complex posttranslational processing, the active enzyme is imbedded in cell membranes and composed of four loosely connected proteins: Aph1, Pen2, glycosylated nicastrin, and endo-proteolyzed presenilin as the catalytic core [13]. γ-Secretase is an aspartic protease [3], [14], with unique preference for some mechanism-based inhibitors [15], unique sequence motifs in the active site [11], [16], and the optimal pH close to the physiological pH [17]. The active site aspartates are located in the central aqueous cavity [18], that can be observed using electron microscopy [19]. The central aqueous cavity is also observed in much smaller intramembrane proteases that have known crystal structures and it could be a result of functionally convergent evolution [11].

Genetics [20], cell biology [2], [10], [12], and drug development studies [21] have indicated that specific changes in enzymatic mechanism of γ-secretase can be enough to trigger development of the disease. FAD mutations (Familial Alzheimer's diseases [20]) can affect more than one third of all amino acids in presenilin 1 (currently about 165 amino acids are listed at www.molgen.ua.ac.be/ADMutations). Different FAD mutations lead to onset of the disease at different age [20], indicating that there are variations in the enzymatic mechanism that make some mutants more prone to the disease than the others. It is unknown how many different enzymatic mechanisms FAD mutations represent, nor whether there is a common enzymatic feature that is shared by the WT and FAD mutants and leads to the development of disease. Apart from FAD mutations, unknown differences in the enzymatic mechanism make Aph1A subunit of γ-secretase more likely to support the pathogenesis than Aph1B subunit [22]. Increase in extent of γ-secretase saturation with its substrate can be a risk factor for development of the disease [23]–[36], possibly due to specific changes in the enzymatic mechanism [37], [38]. Phase III clinical trials showed that γ-secretase inhibitor semagacestat can accelerate the cognitive decline in patients [21]. This serious setback could be a result of the complex inhibition mechanism that shows some features that could facilitate development of the disease [39]–[41].

γ-Secretase has probably more than 50 different substrates, the only substrate linked to Alzheimer's disease is C99, the 99 amino-acid-long C-terminal domain of Amyloid Precursor Protein, APP (APP-C99 [10]). About 25 FAD mutations leading to the disease are found in the C99 sequence (www.molgen.ua.ac.be/ADMutations). The molecular mechanism that makes those mutations pathogenic is unknown. Some FAD mutations are known to affect C99 dimerization [42]–[47]. C99 dimerization correlates with the molecular events associated with the disease, but the actual mechanism is not yet adequately described [42]–[47]. NMR studies showed that C99 substrate is a transmembrane helix [42], with relatively unstructured hydrophilic arms at the C-terminus and the N-terminus. A series of ingenious studies by Ihara and colleagues gave a number of independent lines of evidence that showed that γ-secretase can cleave C99 at multiple sites [37], [40], [48]–[50]. The C-terminal domain is cleaved-off first just underneath the membrane surface. The result is a hydrophobic Aβ fragment and a hydrophilic AICD fragment (Amyloid Intra Cellular Domain). The hydrophobic Aβ fragment is subsequently processively cleaved in steps of three amino acids, to give fragments varying in length from 37 to 49 amino acids [37], [50]. The cleavage sites appear to be interconnected [40], [48], [49], AICD fragment 50–99 will give Aβ fragments 1–49, 1–46, 1–43, 1–40 and 1–37, while AICD fragment 49–99 will give Aβ fragments 1–48, 1–45, 1–42, and 1–38.

A predominant fraction of FAD mutations in C99 substrate is located within Aβ sequence [42]. The disease is often attributed to an increase in Aβ42/Aβ40 ratio that could be a result of “a gain of function for production of Aβ 1–42”, or “a loss of function for production of Aβ 1–40” [20]. Recent studies increasingly show that such debate is an oversimplification [46], [51]. The large amyloid plaques can not be clearly correlated with the pathogenesis [51], so the current research focus is shifted to fibril precursors, most notably unstable oligomers of Aβ peptides [51]–[53]. The oligomerization of Aβ peptides is not an amorphous hydrophobic aggregation [51], [52], [54], [55]. The oligomerization is driven by specific structural forces that have preferred Aβ 1–40/Aβ 1–42 ratio [56]. The oligomer toxicity depends on number of Aβ peptides in the oligomer [53]. Individual Aβ peptides have a highly dynamic structure, varying from α-helix to random-coil to β-sheet [51]–[55], [57]. Such structural fluctuations appear to be crucial for the formation of oligomeric structures and their toxicity [51]–[54], [57], [58]. Furthermore, Aβ 1–43 can be more toxic than Aβ 1–42 in cells and in experimental animals [59], while some cell surface proteins can enhance toxicity of Aβ peptides [60]. Studies of enzymatic mechanism of γ-secretase can greatly advance our ability to understand the toxicity of different Aβ peptides [59], [61].

Surprisingly, very little effort has been invested in attempts to integrate the results from different studies of γ-secretase, its C99 substrate, and its Aβ products into one coherent molecular mechanism. In presented studies we use some standard approaches for studies of enzyme mechanism [62] to analyze WT and two FAD mutations in presenilin 1 of γ-secretase. We trace C99 cleavages from the initial γ-secretase-C99-interaction, to the final release of Aβ product (oligomers). The current knowledge about γ-secretase, its C99 substrate, and its Aβ products is integrated in one coherent molecular mechanism in an attempt to describe pathogenesis of Alzheimer's disease and to propose novel strategies for development of the drug candidates.

## Results

### Catalytic products of γ-secretase can be separated in time

**Figure 1:**
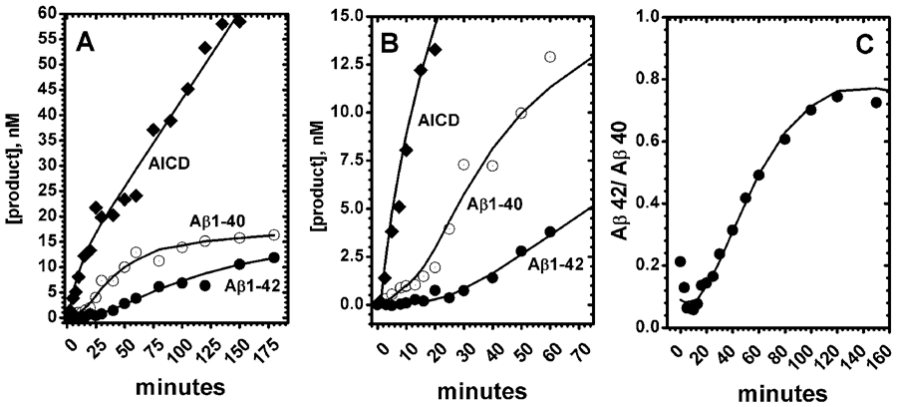
Different phases in γ-secretase reaction can be separated in time. The reactions were prepared using CHAPSO enriched γ-secretase membranes (total protein 0.25 mg/ml) and saturating concentration of C99 substrate (3.0 µM). (A–B). Early time points and the pre-steady-state [62] for AICD, Aβ 1–40 and Aβ 1–42 production (panel B is zoom-in on panel A). The best-fit profile for AICD production was calculated using the equation for pre-steady-state burst (eqn. 1, Table 1), while the best-fit profiles for Aβ 1–40 and Aβ 1–42 production were calculated using the equation for enzyme hysteresis (eqn. 2, Table 1). (C) The time profiles Aβ 1–40 and Aβ 1–42 from figure 1 were used to calculate the changes in Aβ42/Aβ40 ratio as a function of the reaction time (Aβ42/Aβ40 ratio is shown, rather than Aβ40/Aβ42 ratio, in an attempt to follow standards in the literature).

Pre-steady state phase of an enzymatic reaction is routinely used for mapping the order of catalytic steps [62]. Pre-steady state of γ-secretase reaction can be observed by capturing the earliest stage of the first catalytic turnover [62], [63], when AICD, Aβ 1–40 and Aβ 1–42 products initially appear in time (Fig. 1 and Table 1). We find that the C-terminal AICD fragment is produced prior to Aβ 1–40 and Aβ 1–42 fragments in a pre-steady-state burst (Fig. 1 A). The pre-steady-state burst in AICD production indicates that the initial AICD cleavage is fast, and the steady-state rate-limiting step is production and release of different Aβ products (as illustrated in detail in Fig. S1). Y-axis intercept of a pre-steady-state burst can be used to estimate initial concentration of an enzyme-substrate complex (p.p. 156–158 and p. 238 in ref. [62]). The Y-axis intercept for pre-steady-state burst in AICD production indicates that the initial concentration of γ-secretase-C99-complex can be in the range between 5 to 10 nM (Table 1). These values are about 5 to 10 times higher than the values we can measure using the enzyme titration with a highly potent inhibitor LY-411,575 (Fig. S2). As a general principle, the product generated in a pre-steady state burst can be several times higher than the initial concentration of the enzyme-substrate complex if the enzyme can process multiple substrate molecules in one catalytic turnover [62], [64] (i.e. one catalytic turnover consists of multiple catalytic cycles). Thus, we propose that the high burst magnitude is the first out of several lines of evidence that indicates that γ-secretase can bind and cleave multiple C99 molecules in one catalytic turnover.

The time profiles for Aβ 1–40 and Aβ 1–42 production show an initial lag (Fig. 1 A–B). The initial lag for Aβ 1–42 is clearly longer than the lag for Aβ 1–40 (Table 1). This indicates that Aβ 1–40 is produced prior to Aβ 1–42, so that Aβ 1–42 cannot be a precursor for Aβ 1–40. About a dozen of different situations can lead to an early lag in enzyme activity [63]. About a half of them are due to the method of detection, the other half to specific features in the enzymatic mechanism. Duration of the lag for Aβ 1–40 and Aβ 1–42 correlates with the extent of enzyme saturation with its C99 substrate, and traces of the initial lag can be detected in earlier publications that used a different experimental set-up [49], [65], [66]. Calibration of the AlphaScreen® method using synthetic Aβ 1–40 and Aβ 1–42 peptides shows that this method has a linear response well beyond the range measured in the lag. Therefore, the lag is a result of enzymatic mechanism rather than an artifact caused by the measurements. The lag can represent the time period that γ-secretase needs to process the stepwise cleavages of Aβ catalytic intermediates: 1–49. 1–46, 1–43, 1–40 and 1–48, 1–45, 1–42 (Fig. S1). The difference in the length of initial lag (Fig. 1) shows that changes in the enzymatic mechanism that correspond to a shift from Aβ 1–40 to Aβ 1–42 production roughly coincide with the reaction progress from the first to the second turnover (Fig. 1 C). The lowest Aβ42/Aβ40 ratio is observed very early in the lag, i.e. in the early pre-steady-state of the first catalytic turnover (Fig. 1C).

**Table 1:** Kinetic parameters for pre-steady state burst and initial lags (Fig. 1)a, c.

	**AICD a**		**Aβ 1–40 c**	**Aβ 1–42 c**
Intercept nM a	8.2±2	Lag-transition/h c	6±2.4	2±0.6
2σCI b	[6], [13]	2σCI b	[3.1, 12]	[1], [3]
Pre-steady rate/h a	1.2±0.4	Steady-state nM/h	15±1.7	6±0.9
2σCI b	[1.06, 1.75]	2σCI b	[13], [19]	[5.0, 9.6]
Steady-state rate nM/h^a^	21±1.2			
2σCI b	[18], [23]			

a the best fit values ± standard error calculated using a nonlinear regression and the eqn. 1 [69].

b two sigma confidence intervals as described in methods section [69].

c the best fit values ± standard error calculated using a nonlinear regression and the eqn. 2.

**Figure 2:**
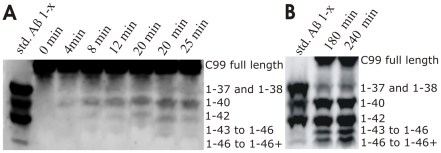
Urea gels show Aβ 1-x products in different phases of γ-secretase reaction. The reactions were prepared using CHAPSO enriched γ-secretase membranes (total protein 0.25 mg/ml), and saturating concentration of C99 substrate (3.0 µM). The lanes “Aβ std 1-x” represents synthetic peptides as mobility standards. To facilitate detection of the early data points **(** A **)** the reaction volume was twenty fold bigger than usual, and the resulting 1-x Aβ products were concentrated about twenty-fold by immunoprecipitation using protein G beads and polyclonal antibodies specific for the first 5 amino acids. It is necessary to mention that pre-incubation of the assay mix for several hours prior to the start of reaction (i.e. addition of C99 substrate) does not affect the relative distribution of different Aβ products. Therefore, the observed changes are not due to enzyme denaturation during the course of the reaction.

We further analyzed the early stage of the reaction using urea gels (Fig. 2 A–B) that can separate Aβ 1–40 and Aβ 1–42 from the other Aβ 1-x products (the urea gels are not as sensitive as the AlphaScreen® measurements). Similar to the AlphaScreen® results (Fig. 1), the urea gels show that Aβ 1–40 dominates the earliest stage of the reaction and that Aβ 1–42 production starts after Aβ 1–40 (Fig. 2 A). The longer more hydrophobic Aβ products are below detection limits in the earliest stage of reaction, and then gradually accumulate with the reaction progress in time. Ultimately, at the late stage of the reaction the longer Aβ products become comparable to Aβ 1–40 and Aβ 1–42 (Fig. 2B). Thus, the longer more hydrophobic Aβ products observed in the late stage of the reaction are not transient catalytic intermediates, but products of an incomplete sequence of the processive cleavages (Fig. S1). This shift to the longer Aβ products can explain the observed drop in Aβ 1–40 and Aβ 1–42 production at the late stage of reaction (Fig. 1). In summary, we conclude that the reaction progress in time can affect the enzyme's ability to process the longer more hydrophobic Aβ peptides to Aβ 1–40 and Aβ 1–42 (different examples of factors that control processing and accumulation of reaction intermediates are illustrated in more details in Fig. S1 and on p.145 in ref. [62]).

### Changes in γ-secretase activity upon saturation with its C99 substrate, small molecule inhibitor DAPT, or NotchΔE substrate

**Figure 3:**
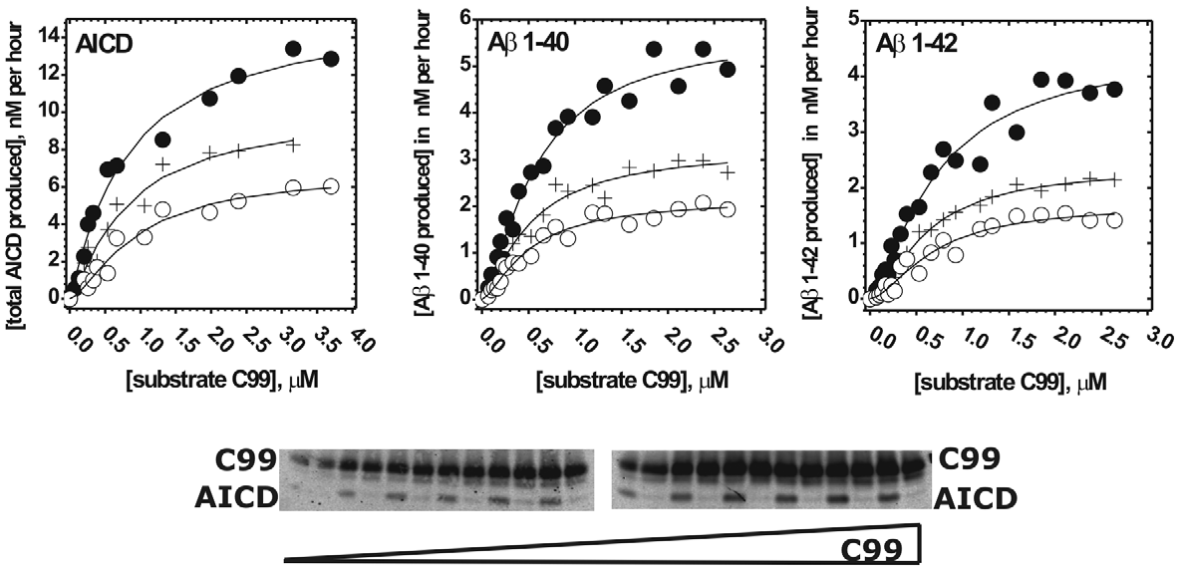
Michaelis-Menten profiles for AICD, Aβ 1–40 and Aβ 1–42 in presence of DAPT. CHAPSO enriched γ-secretase membranes were used to measure Michaelis-Menten profiles for total AICD production in presence of 0 nM (•), 70 nM (+) and 150 nM (O) of DAPT. Michaelis-Menten profiles for Aβ 1–40 and Aβ 1–42 production were measured in presence of 0 nM (•), 100 nM (+) and 200 nM (O) of DAPT. All profiles have been analyzed using nonlinear regression and the eqn. 4 (methods). The corresponding best fit values are summarized in Table 2. The gel strips show different concentrations of the C99 substrate and the corresponding AICD products. Alternating in-between are the parallel control reactions in which γ-secretase was inhibited by a mix of 10 µM of DAPT and LY-411,575 [3], [4]. AICD was measured using antiflag M2 antibodies (as shown in the gel strip). Aβ 1–40, and Aβ 1–42 were measured using AlphaScreen® as described in methods section.

**Figure 4:**
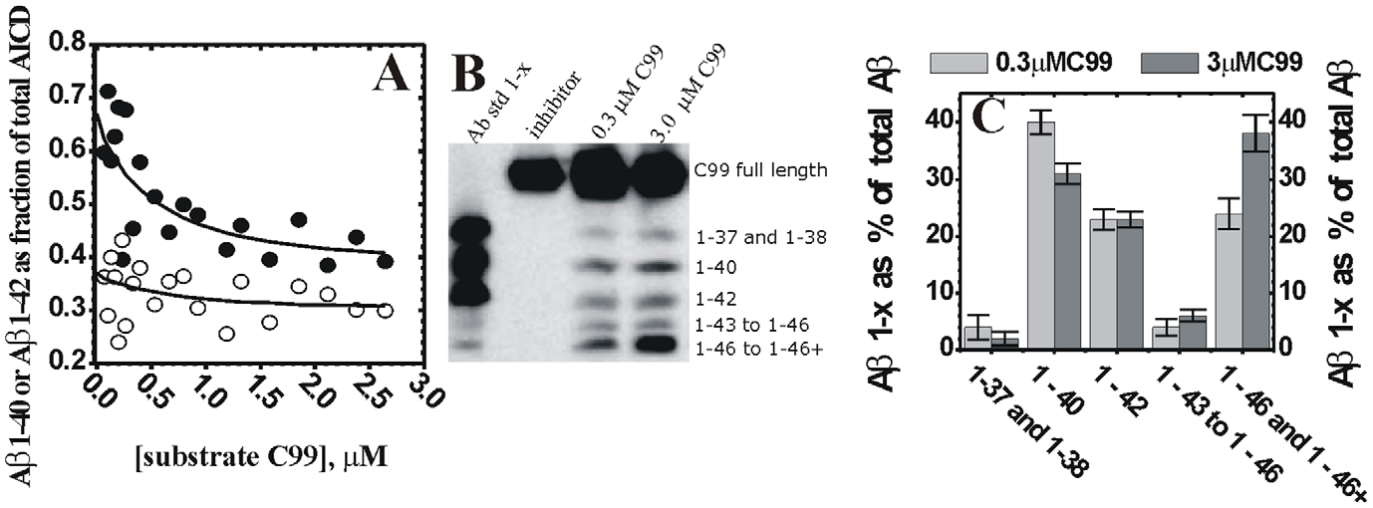
Changes in Aβ products caused by gradual saturation of γ-secretase. Saturation of γ-secretase with its C99 substrate leads to decrease in Aβ40 production with concomitant increase in production of the longer more hydrophobic Aβ peptides and Aβ42/Aβ40 ratio. (A) The saturation profiles from Fig. 3 were used to calculate the ratio between Aβ 1–40 (•) and Aβ 1–42 (O) production and the total AICD production. The ratio curves were calculated using the saturation profiles from Fig. 3 in the absence of DAPT. **(** B **)** Urea gels were used to analyze the relative distribution of different Aβ 1-x fragments at half-saturating (0.3 µM) and saturating (3.0 µM) concentrations of C99 substrate. The lane “Aβ std 1-x” represents synthetic peptides as mobility standards, the lane “inhibitor” represents parallel control reaction in the presence of 10 µM of γ-secretase inhibitors DAPT and LY-411,575 [3], [4]. **(** C **)** The relative intensity of each Aβ 1-x peak is shown as a percent of the total sum of all Aβ peaks in the corresponding lane. The intensity of different Aβ 1-x products was quantified by transforming the individual bands into a series of peaks using the “ribbon option” in program ImmageQuant 5.0. The resulting peaks and the corresponding baselines were quantified using the “peak-fit” option in MicroCal Origin 7.0 program.

**Figure 5:**
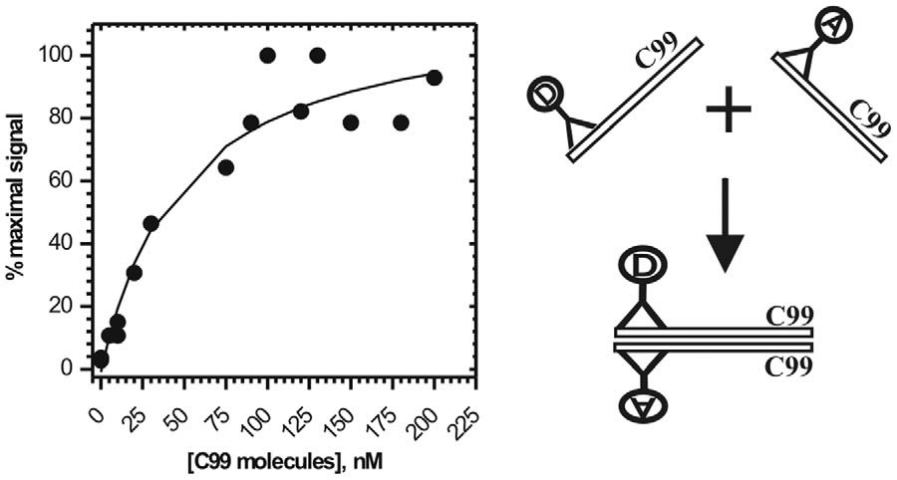
Oligomerization of C99 substrate. C99 dimerization/oligomerization was measured using aliquots of C99 substrate that had high activity with γ-secretase in CHAPSO enriched membranes. Oligomerization between C99 molecules was measured using AlphaScreen® technology by coupling both the donor-beads, and the acceptor-beads, to 3D6 antibody (right panel). Increasing concentration of C99 substrate was incubated with 10 nM of 3D6 monoclonal antibodies coupled to either donor or acceptor-beads. Since one epitope can bind only one antibody, the acceptor and the donor beads can come to proximity and give the AlphaScreen® signal only if C99 dimerization/oligomerization brings the epitopes together (right panel). A nonlinear regression and the equation 5 (methods) were used to calculate an apparent dissociation constant, Kd = 33±2 nM [69].

**Figure 6:**
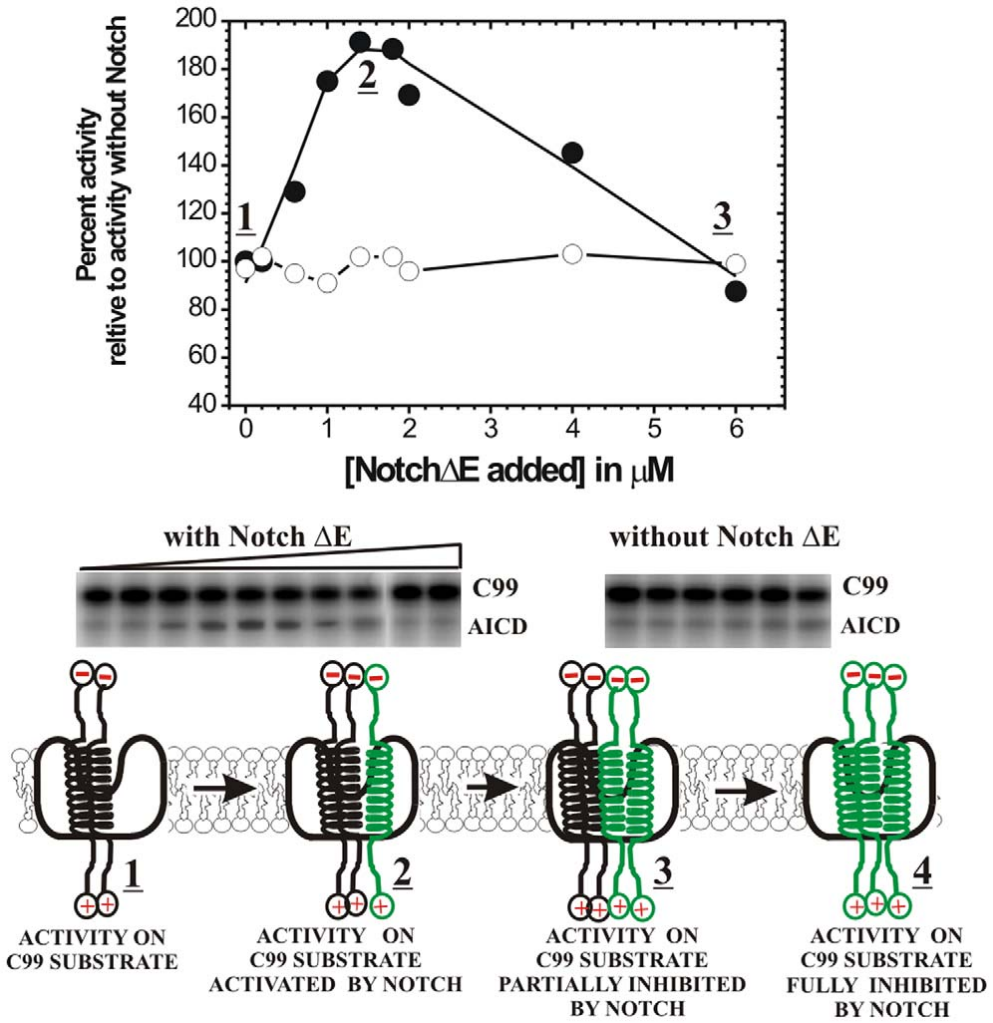
NotchΔE substrate can activate γ-secretase activity on C99 substrate. γ-Secretase activity in CHAPSO enriched membranes was measured using half-saturating C99 substrate ([C99] = 0.45 µM, fresh after purification) in the presence of increasing concentration of NotchΔE substrate (•), and in identical control assays without NotchΔE substrate (O). The AICD production was measured using 125-I labeled C99 substrate and autoradiography as shown on the gel strips (125-I assay was used instead of western blot since both substrates were purified using antiflag M2 epitopes, see methods). Different interactions between γ-secretase and its C99 (black helix) or NotchΔE (green helix) substrates can be illustrated using a model mechanism. C99 substrate can be shown as a transmembrane helix [42], while γ-secretase can be shown as a bowl-shaped membrane-imbedded complex [19]. The underlined numbers connect the different complexes with the corresponding activity range on the graph. In a simple scenario, of one enzyme binding one substrate, NotchΔE and C99 substrates could be only competitive inhibitors [62]. We find that NotchΔE substrate can activate γ-secretase reaction on C99 substrate (**1**). Such scenario can happen only if γ-secretase can bind both substrates at the same time (**2**). NotchΔE substrate shows competition with C99 substrate only when its concentration is several folds higher than C99 concentration (**3**). Extrapolation of the presented profile shows that close to 10 µM of NotchΔE substrate would be needed for a full inhibition (**4**).

Previous studies on humans, experimental animals, cells, and enzymes indicated that increase in the extent of γ-secretase saturation with its C99 substrate can lead to molecular processes that can support the pathogenesis [23]–[35], [37], [38]. We analyze how catalytic mechanism of γ-secretase can be affected by saturation with its C99 substrate (Fig. 3, 4, 5), or small molecule inhibitor DAPT (Fig. 3), or its other substrate NotchΔE (Fig. 6).

**Table 2:** Michaelis-Menten parameters for different γ-secretase products (Fig. 3)^a.^

		AICD^a^			Aβ 1–40^a^			Aβ 1–42^a^	
DAPT	0 nM	70 nM	150 nM	0 nM	100 nM	200 nM	0 nM	100 nM	200 nM

a the best fit values ± standard error were calculated using nonlinear regression and the eqn. 4.

We find that different products of γ-secretase reach saturation at different concentrations of C99 substrate (Fig. 3 and Table 2). Michaelis-Menten constant (Km) for Aβ 1–40 is lower than the constants for AICD or Aβ 1–42 (Table 2). The mechanistic significance of these differences can be revealed by dividing the data points for Aβ 1–40 and Aβ 1–42 with the best-fit Michaelis–Menten curve for total AICD (i.e. the corresponding data points for total AICD) (Fig. 4A and Fig. S3). Such analysis is justified by the fact that every Aβ product has to have one complementary AICD product [37]. We find that at the lowest saturation about 65% of all AICD fragments produced will have one complementary Aβ 1–40, while approximately 30% will have one complementary Aβ 1–42 (Fig. 4A). Intriguingly, with increasing substrate concentrations the relative amount of Aβ 1–40 and Aβ 1–42 product gradually decreased, and the effect is predominately seen on Aβ 1–40 (this is visualized by the steeper descent of the Aβ 1–40/ total-AICD ratio compared to the Aβ 1–42/ total-AICD ratio (Fig. 4A and Fig. S3)). In total, Aβ 1–40/AICD shows about 30% decrease, while Aβ 1–42/AICD shows about 6% decrease at the maximal substrate concentration. Consequently, an increase in C99 substrate results in an increase in Aβ42/Aβ40 ratio. This apparently small 30% decrease in Aβ 1–40 production can have physiological significance since 30% change in Aβ 1–40 metabolism was observed in studies of AD pathogenesis in model organisms [7], [67].

Similar to the results from Michaelis–Menten studies, urea gels show that gradual saturation with C99 substrate leads to decrease in Aβ 1–40 production (Fig. 4 B–C) with concomitant increase in production of the longer more hydrophobic Aβ products (similar experiments is also reproduced in studies of FAD mutations shown later in the text). Different Aβ products in each reaction were quantified by calculating the percentage of each Aβ 1-x product relative to the sum of all Aβ products in the corresponding lane (Fig. 4 C, the same approach was used in similar studies in the past [37]). Relative to the half-saturated reactions (0.3 µM C99), the fully saturated reactions (3 µM C99) shows 15% decrease in Aβ 1–40, no significant changes in Aβ 1–42, 8% increase in Aβ 1–43 to Aβ 1–45, and 15% increase in Aβ 1–46 and Aβ longer than 1–46. The observed changes in Aβ products are smaller than the changes calculated from the Michaelis–Menten analysis, since lower sensitivity of the urea gels did not allow us to use assays with less than 300 nM C99 substrate.

The changes in Aβ products caused by gradual saturation of γ-secretase with its C99 substrate show that the catalytic mechanism is not the same at sub-saturating and saturating substrate [62]. This could be due to: *i*) gradual binding of multiple C99 molecules to γ-secretase; *ii*) C99 dimerization/oligomerization induced by gradual increase in C99 concentration; or *iii*) a combination of those two events. We examine those three possibilities by measuring dimerization/oligomerization of C99 molecules that gave high activity in our assays (Fig. 5). The calculated dimer dissociation constant Kd is equal to 33±2 nM (eqn. 5), which is 10 to 15 fold lower than the Michaelis–Menten constant for Aβ 1–40, Aβ 1–42 and AICD (Table 2). Thus, the shifts in Aβ products shown in figure 4 occur when the majority of C99 molecules are forming dimers/oligomers (the eqn. 5 can be used to calculate the extent of C99 dimerization at different C99 concentrations). In summary, gradual saturation of γ-secretase with its C99 substrate (Fig 3) leads to gradual changes in the Aβ products (Fig. 4) due to gradual increase in the enzyme activity on C99 dimers/oligomers (Fig. 5).

The modulation of γ-secretase activity by multiple enzymes-substrate interactions can be also demonstrated by measuring the enzyme activity with its C99 substrate in the presence of NotchΔE substrate (Fig. 6). In a simple scenario when one enzyme can bind only one substrate, NotchΔE substrate could be only a competitive inhibitor of γ-secretase activity on C99. We find however that NotchΔE substrate can activate γ-secretase reaction on C99 substrate by 85% even when γ-secretase is half-saturated with its C99 substrate ([C99] = 0.44 µM)). Such cooperative effect on the catalytic rates can happen only if both NotchΔE and C99 substrate can bind simultaneously to γ-secretase. NotchΔE substrate starts to inhibit enzymatic reaction on C99 substrate only at higher concentrations. We could not reach sufficiently high concentration of NotchΔE substrate to achieve a full inhibition (the inhibition constant for NotchΔE substrate is expected to be several fold higher than its dissociation constant or its Km constant [68] due to competition with C99 substrate as described on p. 214 in ref. [69]).

We also find that DAPT acts as a noncompetitive inhibitor of γ-secretase when the enzyme is approaching saturation with its C99 substrate (Fig. 3). Thus, at the saturating substrate DAPT and the C99 substrate do not compete for the same binding site on the enzyme.

### Modulation of catalytic activity of γ-secretase by free Aβ products in the reaction mix (Fig. 7)

**Figure 7:**
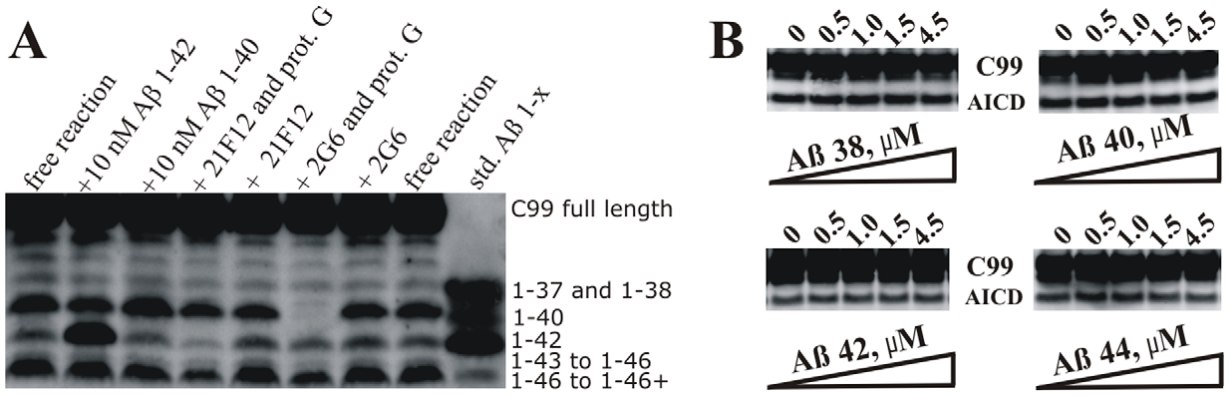
γ-Secretase is not affected by Aβ peptides present in free solution. (A) The lanes labeled as “free reaction” represent Aβ products after 4 hours of routine γ-secretase reaction at half saturating C99 substrate ([C99] = 0.45 µM). The lanes labeled as “+10 nM Aβ 1–42” and “+10 nM Aβ 1–40” represent “free reaction” premixed with synthetic Aβ 1–42 or Aβ 1–40 in a concentration equivalent that corresponds to 4 hours of free reaction. The lanes labeled “+21F12” and “+2G6” represent “free reaction” that was premixed with antibodies specific for Aβ42 or Aβ40 respectively. Both 2G6 and 21F12 antibodies bind the matching Aβ peptides very efficiently as indicated by a complete removal of the corresponding Aβ bands in the reactions with protein G beads (samples labeled as “21F12 and prot. G” and “2G6 and prot. G”). The lane “Aβ std 1-x” represents synthetic peptides as mobility standards. (B) AICD production was measured at half-saturating C99 substrate (0.45 µM) in assays that were premixed with increasing concentrations of synthetic Aβ 1–38, Aβ 1–40, Aβ 1–42, or Aβ 1–44.

Both, the progress of γ-secretase reaction in time (Figs. 1–2) and the gradual saturation with C99 substrate (Fig. 3) result in increase in concentration of different Aβ products in the reaction mix. Thus, there is a possibility that the observed changes in the enzymatic mechanism can be due to Aβ products that (re)associate with γ-secretase and modulate its ongoing catalytic mechanism. We have performed several experiments to test if Aβ peptides present in solution can bind to γ-secretase and affect its catalytic mechanism (Fig. 7).

We find that premixing the reaction mixture with 10 nM synthetic Aβ 1–40 or Aβ 1–42 (Fig. 7A) does not affect the relative difference between Aβ 1–40, Aβ 1–42, and the longer more hydrophobic Aβ products as it can be seen in Fig. 2 and Fig. 4 B. We also find that Aβ 1–40, Aβ 1–42, and the longer Aβ products are not affected when the reaction mix was treated with antibodies specific for the neoepitopes on Aβ 40 or Aβ 42 (by binding to Aβ 40 or Aβ 42 the bulky antibodies could interfere with the repeated interaction between γ-secretase and its Aβ products). Even extremely high concentrations of synthetic Aβ 1–38, Aβ 1–40, Aβ 1–42, or Aβ 1–44 do not affect the rate of AICD production (Fig. 7B, only AICD production could be measured in these experiments since the high concentrations of added synthetic Aβ peptides interfere with Aβ detection in the urea gels).

In summary, we conclude that Aβ peptides present in free solution do not (re)associate with γ-secretase and affect its catalytic mechanism and the Aβ products.

### Comparative analysis of enzymatic mechanism of WT presenilin 1 and FAD mutations G384A and ΔE9

**Figure 8:**
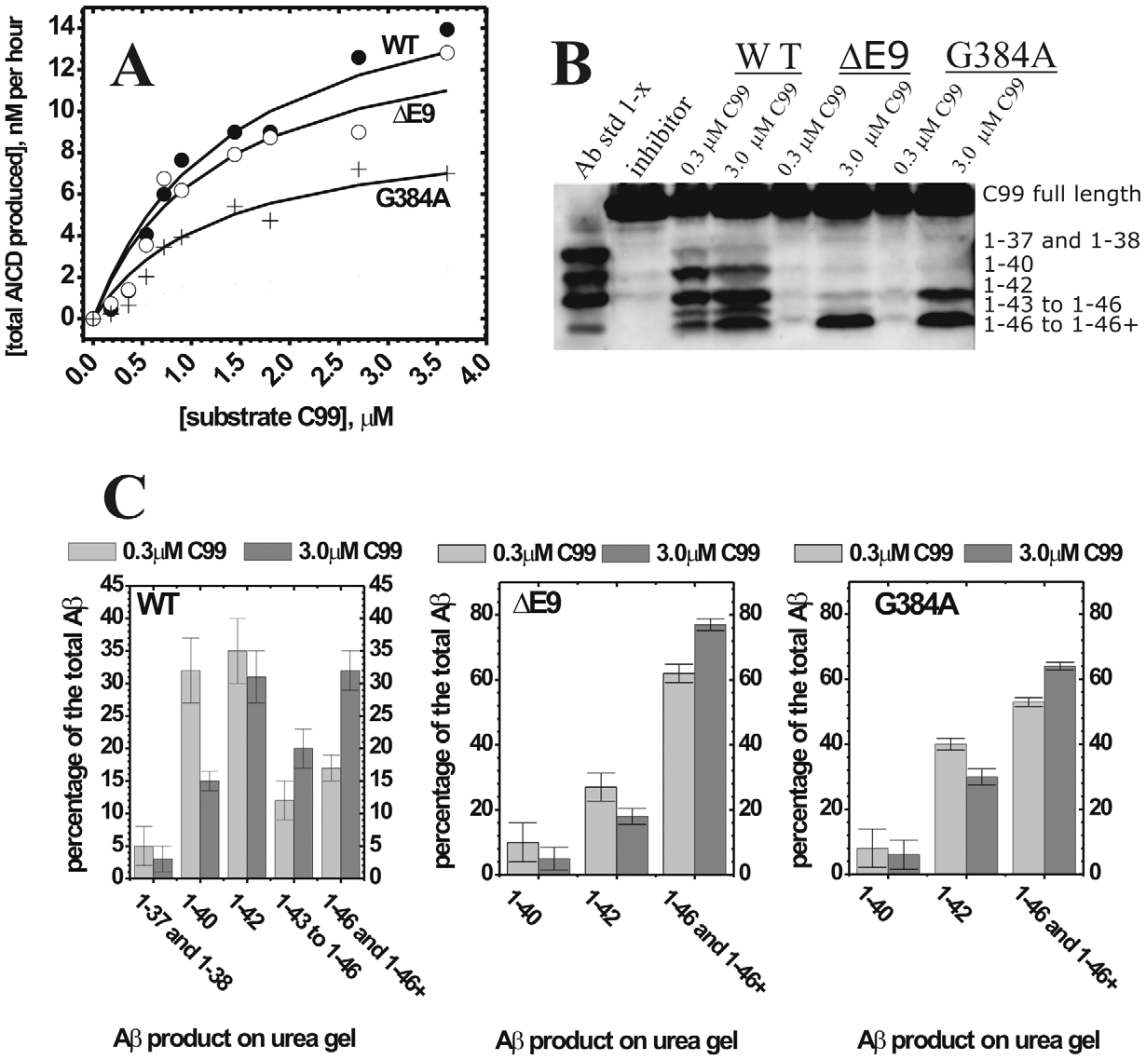
AICD and Aβ production by WT presenilin 1 and two FAD mutants. CHAPSO enriched γ-secretase membranes carrying WT presenilin 1 or FAD mutants ΔE9 and G384A have been prepared and analyzed in parallel with all conditions identical. **(** A **)** Michaelis-Menten profiles for total AICD production (i.e. the turnover rates [37]) were measured in parallel using anti-flag αM2 antibodies as shown in Fig 3. **(** B **)** urea gels show the relative distribution of different Aβ 1-x products at the sub-saturating and saturating substrate concentrations (5 hour reactions). The lane “Aβ std 1-x” represents synthetic peptides as mobility standards, the lane “inhibitor” represents a parallel control reaction in the presence of 10 µM of γ-secretase inhibitors DAPT and LY-411,575 [3], [4] **(** C **)** The relative intensity of each Aβ 1-x peak is shown as a percent of the total sum of all Aβ peaks in the corresponding lane. The intensity of different Aβ 1-x products was quantified by transforming the individual bands into a series of peaks using the “ribbon option” in program ImmageQuant 5.0. The resulting peaks and the corresponding baselines were quantified using the “peak-fit” option in MicroCal Origin 7.0 program.

Comparative analysis of WT presenilin 1 and FAD mutations could highlight changes in the catalytic mechanism that can lead to the pathogenesis. We find that relative to the WT presenilin 1, the total AICD production (i.e. the turnover rates [37]) is about 15% slower for ΔE9 mutation, and about 60% slower for G384A mutation (Fig. 8 A). The Km values for AICD fragments are within experimental error identical (Fig. 8 A). The most significant difference between the WT and the two mutants is in Aβ products (Fig. 8 B–C). To different extent the mutants favor Aβ 1–42 and the longer more hydrophobic Aβ products (Fig 8 B). Different Aβ products in each reaction were quantified by calculating the percentage of each Aβ 1-x product relative to the sum of all Aβ products in the corresponding lane (Fig. 8 C, the same approach was used in similar studies in the past [37]). ΔE9 mutant predominantly generates the longer more hydrophobic Aβ products (i.e. Aβ 1–46 and Aβ 1–46+), while the shorter Aβ products constitute only about 5–10% (Aβ 1–40) and 18–28% (Aβ 1–42) of the total Aβ. Similar to ΔE9 the longer more hydrophobic Aβ products are dominant products with G384A mutant. In difference to ΔE9, Aβ 1–42 is a significant fraction of the total Aβ products with G384A mutant (between 32–40% of the total Aβ). For both mutants Aβ 1–42 stands out as the most dominant short Aβ product (Fig. 8 B). In summary, when compared to the WT presenilin 1, the two FAD mutants show decrease in Aβ 1–40 and increase in the longer more hydrophobic Aβ peptides and Aβ42/Aβ40 ratio.

Due to mutant's low activity in Aβ 1–40 and Aβ 1–42 production, we were unable to achieve the experimental sensitivity that is required for a full quantitative analysis of the Michaelis-Menten profiles as we did with the WT enzyme (Fig. 4A). Nevertheless, the urea gels suggested that for both mutants gradual increase in substrate saturation results in increase in production of the longer more hydrophobic Aβ. The effect appears to be less pronounced than with the WT (Fig. 8 B–C). When fully saturated reaction is compared to half-saturated reaction, ΔE9 shows 5% decrease in Aβ 1–40, 9% decrease in Aβ 1–42, and 15% increase in Aβ 1–46 and Aβ longer than Aβ 1–46. Similarly, G384A shows 4% decrease in Aβ 1–40, 10% decrease in Aβ 1–42, and 11% increase in Aβ 1–46 and Aβ longer than Aβ 1–46. Similar to the data shown in Fig. 4B, WT shows that the saturated reaction has 9% decrease in Aβ 1–40, no significant changes in Aβ 1–42, 5% increase in Aβ 1–43 to Aβ 1–45, and 14% increase in Aβ 1–46 and Aβ longer than Aβ 1–46 (WT lanes in Fig. 8 B–C and Fig. 4 B–C show two independent measurements of the same phenomena).

**Figure 9:**
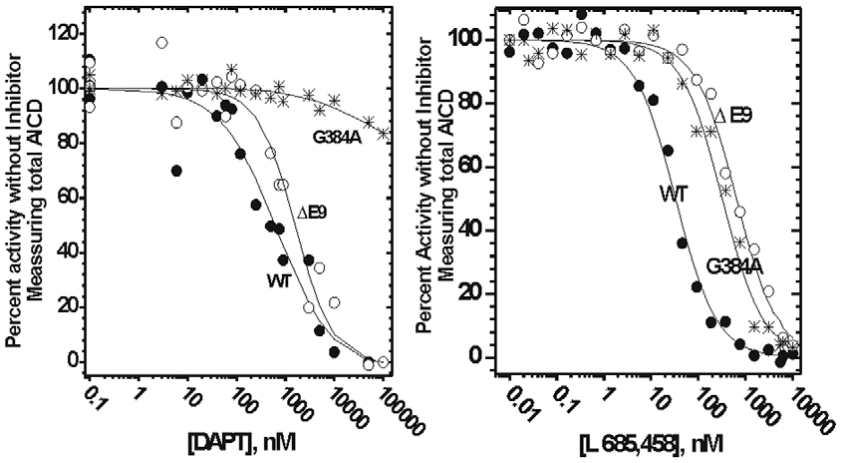
Inhibition of WT presenilin 1 and FAD mutants by DAPT and L-685,458. CHAPSO enriched γ-secretase membranes carrying WT presenilin 1 or FAD mutations ΔE9 and G384A have been prepared and analyzed in parallel with all conditions identical. The dose response curves for DAPT [71] and L-685,458 [15] were measured by following total AICD production using western-blots with αM2 antiflag antibody as shown in Fig. 3. The results were analyzed using nonlinear regression and the equation 3 (methods). The best fit values and the corresponding statistics are given in Table 3.

We used two different classes of γ-secretase inhibitors to analyze how the mutations affect the enzyme structure (Fig. 9 and Table 3). L-685,458 is a transition state inhibitor that is thought to target the active site aspartates [15]. With L-685,458, ΔE9 and G384A show similar 10–20 fold decrease in inhibition potency relative to the WT (Fig. 9 and Table 3). Such decrease in IC50 values can be a result of a loss in binding energy equivalent of one misplaced hydrogen bond ([62], some illustrative examples can be found in ref. [15]). Thus, the two mutations result in similar and relatively small perturbations in the active site structure. Very different situation is observed with DAPT, an inhibitor that is targeting N-terminal of presenilin 1 in the transmembrane domain 7 [3]. With DAPT, ΔE9 mutation shows approximately twofold decrease in inhibition potency relative to the WT (Table 3), while G384A mutation shows about 1000-fold decrease in the inhibition potency and a low (shallow) Hill's coefficient [69] (Fig. 9, Table 3). The low Hill's coefficient [69] indicates that the mutation leads to structural heterogeneity (i.e. constrained flexibility) at the DAPT binding site, and/or a binding antagonism with the C99 substrate [69]. Finally, IC50 values for both DAPT and L-685,458 with the WT enzyme are very similar to the values measured in cell-based assays [3], [15]. Thus, the WT enzyme has very likely same structure around the inhibitors' binding sites in our enzyme-based assays and in the previous cell-based assays [3], [15].

**Table 3:** Inhibition of WT presenilin 1 and FAD mutants by (Fig. 9)^a.^

Inhibitor:	DAPT				L-685,458	
Presenilin 1:	WT	ΔE9	G384A	WT	ΔE9	G384A
**IC50,nM** a	390±179	734±254	3 10^6^±1·10^6^	32±4	707±80	398±51
**2σCI** b	[150, 540]	[505, 934]	n.a. c	[29], [35]	[631, 776]	[339, 479]
**Hill's coef.** a	0.7±0.2	1.21±0.07	0.54±0.25	1.3±0.1	0.98± 0.08	1±0.13
**2σCI** b	[0.55, 1]	[1.54, 1.07]	n.a. c	[1.1, 1.4]	[0.9, 1.06]	[0.86, 1.14]

a the best fit values ± standard error were calculated using nonlinear regression and the eqn. 3.

b two sigma confidence intervals as indicated in methods section [69].

c cannot be calculated due to the limited data range.

## Discussion

**Figure 10:**
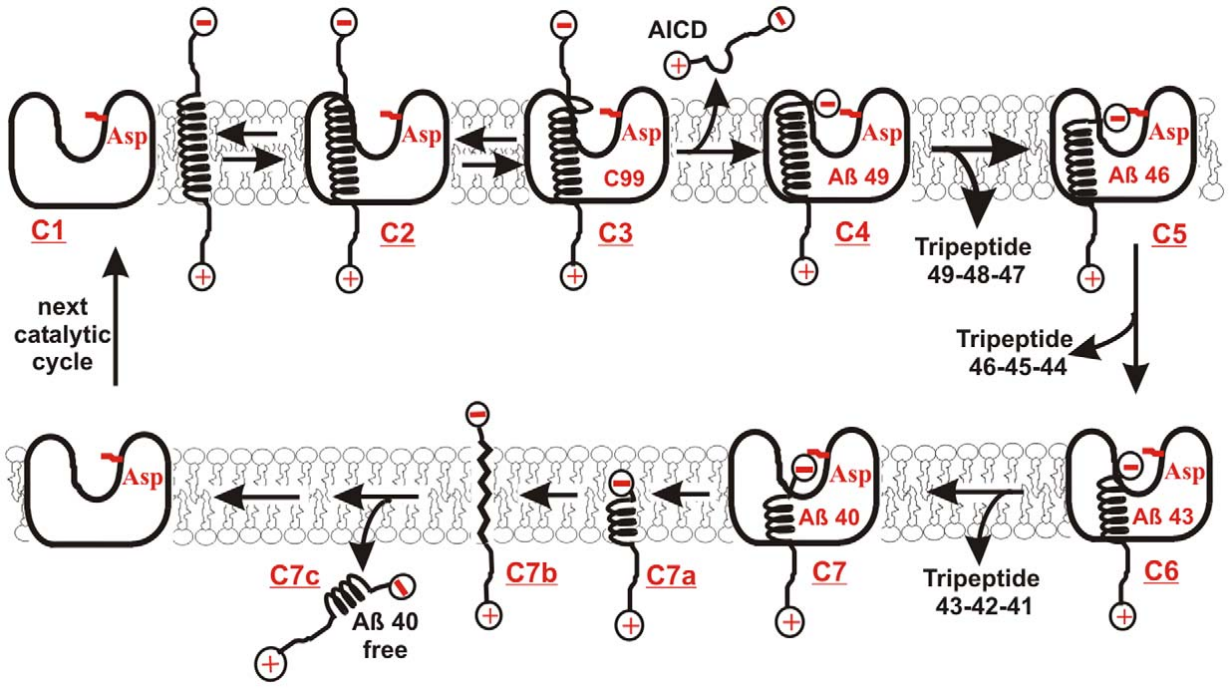
**Steps in the catalytic cycle of γ-secretase.** The model illustrates the basic biophysical principles of processive cleavages and intramembrane proteolysis [11], [37], [40], [48], [49], [51]. C99 substrate can be shown as a transmembrane helix [42], while γ-secretase can be shown as a bowl-shaped membrane-imbedded complex with its active site aspartates in the central aqueous cavity [11], [18], [19]. The initial AICD cleavage (Fig. 1) takes place between amino acids 48–49 or 49–50 [37], just under the membrane surface [42], in a dynamic section that has a tendency to destabilize the transmembrane helix ((C1->C4), [58]). The result is a soluble AICD fragment, and a hydrophobic Aβ fragment with its negatively charged carboxyl-terminal trapped below the membrane surface (C3->C4). Thus, the negatively charged carboxyl-terminal is in an energy gap that is forcing it to the interface between the hydrophobic enzyme core and the hydrophilic central aqueous cavity. The opposing force comes from the hydrogen bonds that tend to stabilize the transmembrane helix (C4). The Aβ peptides have a highly dynamic structure that can vary from α-helix to random-coil [51]–[55], [57]. Such structural changes can drag small parts of the hydrophobic Aβ peptides to the active site aspartates following the negatively charged carboxyl-terminus in the central aqueous cavity ((C4->C7), [11]). Thus, the whole process can be driven by entropy and/or by repulsive forces between negative charges on the active site aspartates and the carboxyl-terminal on the nascent Aβ [51]–[54], [57]. There is no need for active use of cell's energy. The result is a sequence of processive cleavages of hydrophobic tri-peptides [48] that does not require a full exposure of the hydrophobic substrate to the aqueous catalytic site [11]. The initial cleavage at 49–50 site leads to Aβ 49–46–43–40 sequence, while the initial cleavage at 48–49 site leads to Aβ 48–45–42–38 sequence [37], [40], [48], [49]. It is very important to realize that the most frequent end-products Aβ 1–40 and Aβ 1–42 have more than a half of the original hydrophobic transmembrane helix of C99 (C6->C7). Such products are highly unlikely to spontaneously dissociate from the hydrophobic γ-secretase to the hydrophilic extracellular space (C7c). Furthermore, the peptides are too short to form a transmembrane helix (C7a) [62], while the fully extended structures (C7b) can not be stabile due to unsatisfied hydrogen bonds in the peptide backbone [62]. For the same reasons the nascent Aβ-peptides (C1->C6) can not be spontaneously released from γ-secretase. The hydrophobic Aβ products can dissociate from γ-secretase only by interacting with a carrier protein, or by forming an Aβ bundle as in Fig. 11. The carrier protein is expected to facilitate catalytic rates since dissociation of Aβ products is the rate-limiting step (Fig. 1, and Fig. S1). Thus, possible candidates for the carrier protein can be the proteins identified by He and coauthors [93], apo-lipoprotein E [5], PrP C [94], or some other surface proteins [60].

**Figure 11:**
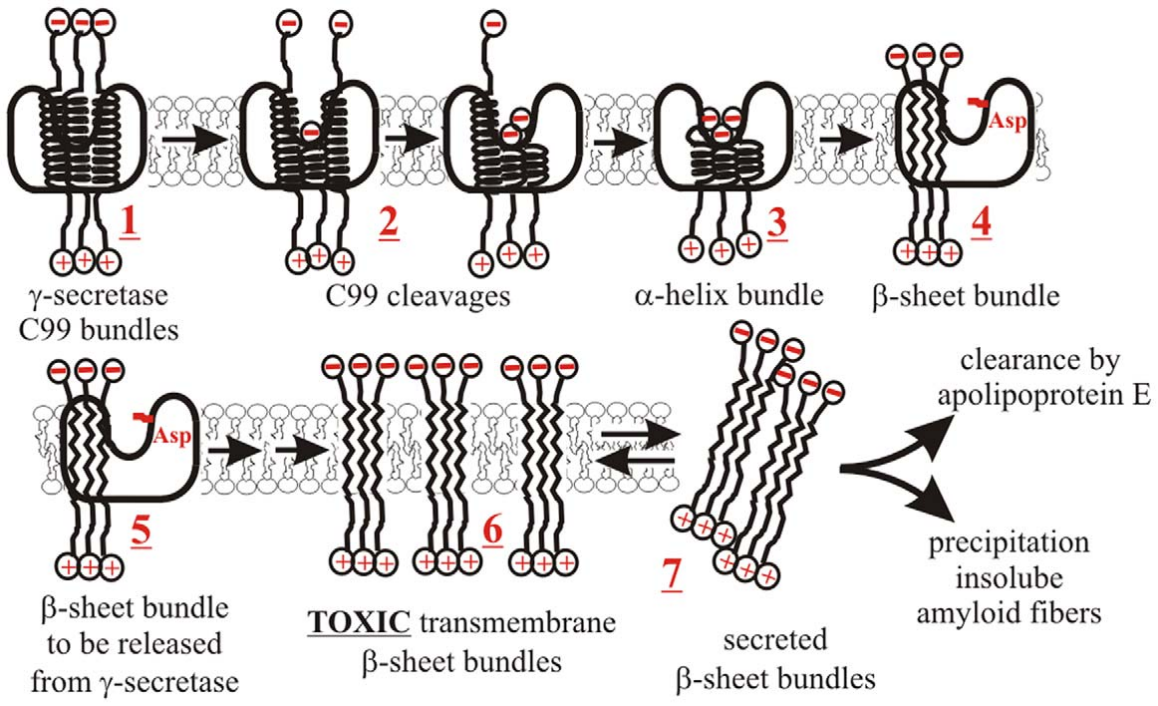
Multiple C99 molecules bound to γ-secretase can facilitate the pathogenesis. Multiple C99 molecules bound to γ-secretase can affect the catalytic mechanism and contribute to the neurotoxic events. Multiple C99 molecules bound to the enzyme (1) could interact just as free C99 molecules [42]–[46]. Such interactions can influence the initial AICD cleavage and thus control the difference between Aβ 49–46–43–40 or Aβ 48–45–42 cleavage paths (Fig. 10). If multiple C99 molecules are cleaved in parallel, the result will be a bundle of nascent Aβ peptides (3), or even a mixed bundle of C99 and nascent Aβ peptides (2). All of those interactions can be affected by the same structural forces that control interactions between Aβ peptides in free solution. Thus, there could be a preferred number of peptides in the bundle [52], [53], and a preferred ratio between Aβ40, Aβ42, and the longer Aβ peptides [56]. Any of those can affect dynamic structural changes that control the processive cleavages, and ultimately the type of Aβ products (Fig. 10). Packed together the nascent Aβ peptides can undergo a series of structural changes so that their β-genic amino acids (Thr, Val, Ile) can initiate formation of extended β-sheet bundles (3->4) [51]–[54], [57], [58]. This can drive transition from the α-helix structure of C99 to the β-sheet structure of Aβ oligomers [51]–[54], [57], [58]. The whole process can be chaperoned and accelerated by the enclosure within the enzyme structure. Some functional and evolutional links have been observed between chaperones and rhomboid intramembrane proteases [95], [96]. Unlike single amyloid peptides (Fig. 10), the hydrophobic β-sheet bundles can be easily released into the lipid bilayer (5–>6). The bundles can be stabilized by hydrogen bonding between the peptides' backbones so that their hydrophobic amino acids can face the lipid bilayer [51]. The released β-sheet bundles can accumulate to toxic levels by causing disruption of membrane integrity (i.e. fluidity, lipid rafts and ion gradients [51], [97]). Thus, the neurotoxic processes can start directly in the membrane where toxic amyloid peptides are produced, rather than in the extracellular space as it was suggested in the original amyloid hypothesis and its subsequent derivatives [51]. Extracellular amyloid fibrils can be the end result of chronic toxic overload and the final membrane breakdown (**7**) [51].

There is a standing debate whether pathological increase in Aβ42/Aβ40 ratio is a result of “a gain of function for production of Aβ42”, or “a loss of function for production of Aβ40” [20]. We find that increase in Aβ42/Aβ40 ratio can be caused by: *i*) increase in Aβ 1–42 production due to progress of γ-secretase reaction from pre-steady-state to steady-state catalysis (Fig. 1 and 2), *ii*) decrease in Aβ 1–40 production due to enzyme saturation with its C99 substrates (Fig. 3–4 and Fig. S3). In both cases, increase in Aβ42/Aβ40 ratio and decrease in Aβ40 production correlates with increase in production of the longer more hydrophobic Aβ products. The presented results are consistent with the earlier studies [37], [38], [40], [48]–[50]. The molecular mechanisms that can lead to such changes are elaborated in detail in Fig. 10 and Fig. 11. The increase in Aβ42 production can be attributed to changes in γ-secretase-C99 interaction, so that the initial cleavage takes place between the amino acids 48–49 rather than between 49–50 (Fig. 10). The increase in the longer more hydrophobic Aβ products can be attributed to decreased ability of γ-secretase to hold and fully process the nascent Aβ catalytic intermediates (Fig. 10 and Fig. 11).

The idea that γ-secretase can bind more than one C99 molecule was presented many times in the past. It has been proposed that the substrate can translocate from a docking site to the active site [66], [70], [71], or that the enzyme has a regulatory allosteric site and the catalytic site [72], [73]. Here we present four different lines of evidence that γ-secretase can bind and cleave multiple substrate molecules in one catalytic turnover. Namely, *i*) gradual saturation with C99 substrate leads to changes in the enzyme mechanism (Fig. 4); *ii*) the enzyme shows high activity with substrate dimmers/oligomers (Fig. 5); *iii*) C99 cleavage can be activated by NotchΔE substrate (Fig. 6); *iv*) high magnitude of the pre-steady state burst (Fig. 1 and Fig. S2). Several studies showed that γ-secretase can cleave C99 dimers [42]–[45], including C99 molecules covalently attached to dimers [46]. Therefore, the substrate binding cavity must be large enough to accommodate more than one C99 molecule (Fig. 11). We propose that binding of multiple C99 molecules into one active site cavity (Fig. 11) is the most straightforward explanation for the studies that proposed multiple binding sites [66], [70]–[73], showed cleaving of C99 dimers [42]–[46], and the present results (Fig. 1, 2, 3, 4, 5, 6).

Increase in Aβ42/Aβ40 ratio, and increase in production of the longer more hydrophobic Aβ products appears to be a shared feature between different conditions that could support development of the disease. To different extent, both of these features can be observed: *i*) when γ-secretase is saturated with it C99 substrate (Fig. 4, and Fig. S3); *ii*) when ΔE9 and G384A FAD mutations are compared to WT presenilin 1 (Fig. 8B); and *iii*) when Aph1A subunit is compared to Aph1B subunit of γ-secretase [22]. Aβ 1–43 can be more toxic than Aβ 1–42 in model organisms and in cells [59]. Our ability to explore pathophysiology of Aβ products longer than Aβ 1–42 is in a large part limited by our ability to understand the enzymatic mechanism that leads to their formation [59], [61]. The longer Aβ peptides are highly hydrophobic, difficult to measure, and only a small fraction of reported studies have met the experimental challenges [22], [37], [40], [48]–[50]. Nevertheless the longer Aβ peptides are catalytic intermediates that can give valuable insights to the pathogenesis [59], [61] and the catalytic mechanism (Fig. 10). Studies of the longer Aβ can also provide answers to many of the earlier confusions that came from studies that rely only on measurements of Aβ42, Aβ40, Aβ38 and/or Aβ42/Aβ40 ratio [46], [48], [61]. The longer more hydrophobic Aβ products can also explain why forced dimerization of C99 substrate leads to decrease in the secreted Aβ products and increase in AICD production [46].

Quantitative studies of the enzyme mechanism are possible only in enzyme-based assays that allow control of the reaction time (Figs. 1 and 2), and the extent of enzyme saturation with its different ligands (Figs. 3, 4, 5, 6, 7 and 9) [62]. The enzyme-based assays can be correlated with cell-based assays. For healthy cells the most frequently quoted value for Aβ42/Aβ40 ratio is 1∶10 [1], [48]. Our enzyme-based assays show that the closest similarity with the cell-based assays can be achieved at the lowest saturation with C99 substrate (Fig. 4A), and in the early pre-steady-state (i.e. the first 10 minutes of reaction) (Fig. 1–2). This is not surprising, since low saturation and pre-steady state conditions are closest to the general conditions that exist in cells [74]–[76]. In cells enzymes and substrates are present in low saturation and in similar concentrations [74]–[76] (we have developed experiments that show that γ-secretase is far below saturation in cells, a manuscript is in preparation). Such setting is the most suitable for fine tuning of cell physiology since even the smallest change in any parameter can give a direct response from the related parameters [62], [74]. In the future we have to increase the sensitivity of our assay to improve measurements at low saturation (<100 nM) and in pre-steady-state conditions (<8 min). Such strategy can allow us to address other potential concerns about the differences between cell-based and enzyme-based assays [48], but also to correlate pre-steady state studies of γ-secretase (Figs. 1, 2, 3, 4) with the biophysical studies of C99 and different Aβ peptides [47], [51]–[55], [57].

Relatively high C99 concentration is needed to saturate γ-secretase in the enzyme-based assays (Fig. 3 and [22], [37], [38]) since formation of the enzyme-substrate complex depends on free-diffusion in three-dimensions in a highly diluted protein solution (0.25 mg/ml). In cells, γ-secretase and C99 molecules are constrained in two-dimensional membranes, most likely in narrow membrane rafts [77] and multi-molecular complexes [78], in a medium with extremely high protein concentration (>200 mg/ml, [79], [80]). Both, the limited diffusion and the molecular crowding effects can facilitate the component's association rates and the interaction energy by several orders of magnitude [79], [80]. Thus in cells the enzyme-substrate complex is formed under influence of local C99 concentrations [79], [80], that cannot be directly compared with C99 concentrations in whole cell-extracts or the enzyme-based assays (Fig. 3). Nevertheless, different enzyme-based studies (Fig. 4 and [37], [38]), and different studies on humans, experimental animals, and cells have shown that in all cases gradual saturation of γ-secretase leads to molecular events that have been associated with the pathogenesis [23]–[35].

C99 dimerization/oligomerization has been observed in cells over-expressing C99, and with purified C99 [42]–[45], [47]. It remains unknown to what extent endogenous C99 substrate is dimerized/oligomerized in healthy cells [81]. Since dimerization affects Aβ42/Aβ40 ratio [42]–[46] and other physiological processes [8], [81]–[83] it can be expected that the cells have developed some physiological mechanisms that control C99 dimerization. Cell-free assays do not have the physiological processes that can prevent C99 dimerization (Fig. 5), however different dilutions of C99 substrate represent different extent of enzyme saturation with C99 dimers/oligomers (eqn. 5, methods).

Comparisons of WT presenilin 1 with ΔE9 and G384A FAD mutants (Fig. 8–9) gave us a glimpse into structural changes that could lead to the pathogenic changes in Aβ products. G384A and ΔE9 FAD mutations were chosen as two mutations that could have very different effect on the enzyme structure around the two active site aspartates [13]. G384A is a mutation in a highly conserved active site loop GXGD next to the active site aspartate D385 [11], [13], [16]. This apparently subtle change is the only mutation at that position that can give an active enzyme [11], [13], [16]. ΔE9 is a mutation at a splice acceptor site that results in a deletion of a link between the two transmembrane helixes that carry the active site aspartates (amino acids 290–319 [13], [84]). ΔE9 mutation appears to be less pathogenic than G384A. ΔE9 leads to onset of Alzheimer's disease at an average age of 45.5, with death at an average age of 51.2 [85]. G384A leads to onset of Alzheimer's disease at an average age of 34.9, with death at an average age of 42.2 [85].

The two mutations have relatively small effects on the total turnover rates (Fig. 8A), and the structure around the active site aspartates (Fig. 9 and Table 3). The most significant difference relative to the WT is in distribution of different Aβ products (Fig. 8 B–C). High prevalence of the longer Aβ products indicate that the two mutations affect the enzyme's ability to hold the nascent Aβ catalytic intermediates before they can be fully processed to the shorter Aβ products (Fig. 10 and Fig. 11). Relatively large fraction of Aβ42 indicates that G384A mutation specifically supports structural changes that favor amino acids 48–49 as the initial cleavage site (Fig. 10). Surprisingly, G384A mutation next to the active site aspartates D385 has bigger effect on the inhibitor targeting the N-terminal section of transmembrane 7, than on the inhibitor targeting the active site aspartates (Fig. 9 and Table 3). The surprising difference in sensitivity to different classes of γ-secretase inhibitors indicates that G384A mutation is not a local mutation in the highly conserved active site loop [11], [16]. More likely scenario is the proposal that G384A mutation can disrupt the sliding interactions between the transmembrane helixes 6 and 7 [11], [16]. In summary, our results suggest that FAD mutations primarily affect the enzyme's interaction with the nascent Aβ catalytic intermediates and C99 substrate (Fig. 10), while there is relatively little effect on the active site aspartates.

We can use the presented conclusions to contemplate about mechanism of action for known inhibitors of γ-secretase and about possible alternative drug-design strategies [3], [4]. Based on presented arguments a successive therapy needs to decrease the extent of enzyme saturation with its C99 substrate. Thus, an effective drug would be a compound that will increase the Km for C99 substrate with a minimal effect on the turnover rate for Aβ 1–40; i.e. a standard competitive inhibitor for Aβ 1–40 [62]. Noncompetitive inhibitors such as DAPT (Fig. 3) can have exactly opposite effect from desired. Noncompetitive inhibitors will lead to decrease in enzyme catalytic capacity, which will make the enzyme saturated even at the lower levels of its C99 substrate (remember that maximal activity is equal to the total enzyme concentration multiplied by its turnover rate, p.p. 105–109 in [62]). Consistent with the presented proposal, different genetic manipulations have shown an increase in Aβ42/Aβ40 ratio when the total catalytic capacity of γ-secretase in cells is decreased [33], [35], while the opposite effects are observed when the enzyme catalytic capacity is increased [34]. The ability of noncompetitive inhibitors to facilitate the progress of Alzheimer's diseases also depends on its pharmacokinetics and pharmacodynamics properties. Phase III clinical trials on 2600 patients showed that semagacestat can facilitate cognitive decline that is characteristic for the disease [21]. Preclinical studies of semagacestat have never been released [86]. However there is a good possibility that semagacestat is a noncompetitive inhibitor just like DAPT (Fig. 3) based on the structural [3], [4] and the functional similarities [39]–[41].

In the future all γ-secretase inhibitors should be tested in the enzyme-based studies to avoid unnecessary harm to patients and costly failures in clinical trials. The key criteria in screening for effective leads should be competitive inhibition and preservation of Aβ40/AICD ratio (Fig. 4). The two screening criteria should be selective for favorable Aβ42/Aβ40 ratios, the short Aβ products (Fig. 4), and the preserved functioning of different signaling pathways [3], [4]. The proposed strategy is encouraged by the observations that the same disease promoting changes in the Aβ products come with very different changes in the total AICD production (which is equal to the total enzyme activity). Saturation of WT γ-secretase with its C99 substrate leads to an increase in total enzyme activity and AICD production (Fig. 3), while FAD mutations lead to a decrease in total enzyme activity and AICD production (Fig. 8). γ-Secretase complex containing Aph1A subunit shows pathogenic changes in Aβ products relative to Aph1B complex with almost no difference in AICD activity [22].

### Conclusions

We propose that gradual saturation of γ-secretase with its substrate can be the pathogenic process in different alleged causes of Alzheimer's disease (Fig. 11). Studies on humans, experimental animals, and cells described some of the conditions that can lead to gradual saturation of γ-secretase and the pathogenesis. Namely: *i*) increased expression of the APP gene [28]–[30], or any other increase in APP metabolism [8], [36]; *ii*) increased activity of β-secretase [23]–[27], or the Swedish mutation in the APP sequence [31], [32]; *iii*) changes in the expression of active γ-secretase [33]–[36]; *iv*) insufficient clearance of Aβ products [7], [36]. This list is likely to grow in the future as we learn more about the factors that control APP metabolism [8], [36]. Saturation can be induced even at normally sub-saturating substrate if the enzyme is exposed to noncompetitive inhibitors such as DAPT (Fig. 3) [3], [4], or to its alternative substrates such as NotchΔE (Fig. 6)

## Materials and Methods

### Cell cultures

Cos1 cells were obtained from ATCC, while MEF (mouse embryonic fibroblasts) cells were obtained from the previous studies [18]. The cells were grown in DMEM media (Invitrogen) supplemented with 10% fetal calf serum (Sigma). All cell cultures were propagated by reseeding the cells every three days using 1% trypsin (Sigma).

### Materials

Antibodies used in these studies were: 82E1 (Takara BIO, cat. number 10323) prepared to recognize the first 16 N terminal amino acids on human C99 or Aβ fragments [50]. 3D6, prepared against the first 6 N-terminal amino acids in human C99 or Aβ fragments [87]; 2G3, a monoclonal antibody that reacts strongly with Aβ40 but has essentially no cross-reactivity with Aβ42 [88], 21F12, a monoclonal antibody that reacts strongly with Aβ42 but has essentially no cross-reactivity with Aβ40 [87]. Anti-flag αM2 monoclonal were purchased from Sigma-Aldrich (product number F2555).

γ-Secretase inhibitors DAPT (*N*-[*N*-(3,5-difluorophenacetyl)-L-alanyl]-*S*-phenylglycine *t*-butyl ester) and L-685,485 ({1*S*-benzyl-4*R*-[1*S*-carbamoyl-2-phenylethylcarbamoyl-1*S*-3-methylbutylcarbamoyl]-2*R*-hydroxy-5-phenylpentyl} carbamic acid *tert*-butyl ester) were purchased from Calbiochem. CHAPSO (3-[(3-cholamidopropyl)dimethylammonio]-2-hydroxy-1-propanesulfonic acid) used in these studies was always kept on 4°C, and its shelf life was never longer than six months. Bicine (2-(Bis(2-hydroxyethyl)amino)acetic acid), PIPES (1,4-piperazinediethanesulfonic acid), Tricine (*N*-[2-hydroxy-1,1-bis(hydroxymethyl)ethyl]glycine), and Tween 20 (Polyoxyethylene (20) sorbitan monolaurate) were purchased from SigmaAldrich.

### Preparation of C99 substrate and NotchΔE substrate

Both human C99 and human NotchΔE substrates were prepared as earlier described [22], [89]. Briefly, COS1 cells were transiently transfected with pSG5 vector (plasmid Stratagene, SV40 early promoter) carrying C99 or NotchΔE sequences with 3xFLAG sequence at its C-terminus. Fifteen hours prior to harvest the cells were treated with 10 µM of γ-secretase inhibitor GM6001 (CalBiochem, cat. # 364206) to prevent production of C83-3xFLAG. The scraped cells were re-suspended in 50 mM Tris-HCl, pH 7.6, 150 mM NaCl, 1% Nonidet P-40 (NP40 (IgepalCA-630): Sigma), plus complete protease inhibitor mixture (Roche) and incubated on ice for 1 h. Membrane-solubilized protein fractions were obtained by ultracentrifugation at 245,000×*g* for 20 min. Immunoaffinity purification was carried out with the anti-FLAG M2-agarose beads (Sigma), according to the manufacturer's protocols. APP C99-3×FLAG was eluted in 100 mM glycine HCl, pH 2.7, 0.25% *n*-dodecyl β-D-maltoside (Sigma) and immediately neutralized to pH = 7 by adding 1M Tris-HCl, pH = 8.0. The final substrate concentration was determined based on i) A280 absorbance and calculated extinction coefficient 5.96 10^3^M^-1^ cm^–1^, and ii) based on BioRad Bradford reagent with correction for BSA standard as indicated by the manufacturer. The two methods give within experimental error consistent results. For 125-I assays, Perkin-Elmer Iodogen kits were used to label 500 µl of 1 µM of fresh purified C99 with 1 µCi 125-Iodine in 30 minutes. Labeled C99 molecules were separated from free 125-I using PerkinElmer PD 10 columns. The labeled C99 was concentrated and used immediately in γ-secretase assays.

### Preparation of cell membranes with γ-secretase (i.e. microsomal fractions)

MEF cells, or MEF double knockout for endogenous presenilin transduced with human WT, dE9 and G384A presenilin 1 [85], were grown to confluence, scraped, and collected in pellets by centrifugation at 1000×g for 5 min. The cell pellets were re-suspended in 20 mM Pipes pH = 7.0, 140 mM KCl, 0.25 M sucrose, 5 mM EGTA, plus 1X Roche protease inhibitors cocktail, so that the total protein concentration was 10 mg/ml. Re-suspended cells were subjected to more than 20 passages in 8.010 mm cell-cracker. The resulting cell extract was subjected to 10 min centrifugation on 2000×g to remove large debris, and the fragmented membranes were collected as pellets after centrifugation for 1 hour at 100 000×g, and stored at −80°C.

### γ-Secretase activity assays using CHAPSO enriched membranes

γ-Secretase assays using CHAPSO enriched membranes were performed essentially as earlier described [22], [37], [48], [89]. Briefly, microsomal fractions (protein concentration 10 mg/ml) from different MEF cells were solubilized in 1% CHAPSO buffer (50 mM Pipes, pH 7.0, 0.25 M sucrose, 1 mM EGTA, 1×Complete protease inhibitor mixture (Roche)) and incubated on ice for 1 h. CHAPSO was always prepared as 1% w/v fresh from a powder stock that was less than 6 months old and kept at 4°C (freshness is crucial for high activity). Next, the membrane-solubilized protein fractions were obtained as supernatant by ultracentrifugation for 1 hour at 100,000×*g*. The prepared CHAPSO enriched membranes were diluted two fold with 50 mM Pipes pH = 7.0, 0.25 M sucrose, 1 mM EGTA, 0.1% phosphatidylcholine, and 0.0125% phosphatidylethanolamine, plus 1×Complete protease inhibitor mixture (Roche), and left on 37°C for two to three hours. This incubation can increase the measured activity by up to 70% since it can accommodate slow reassembly of γ-secretase components that is induced by the transition from 1% CHAPSO to 0.25% CHAPSO [90]. The reactions were started by adding C99 substrate in desired concentration, the added volume was adjusted so that: i) the final CHAPSO concentration was 0.25%; ii) and final concentration of membrane proteins was 0.25 mg/ml. Fresh C99 substrate that is used immediately after purification gives the best opportunity to observe described enzymatic features and the highest activity. The assay mix was prepared in low adhesion microcentrifuge tubes, the volume was usually 25 µL. To increase sensitivity in early data points detection, and at low enzyme saturation, the assay volume was increased up to 400 µL, and the resulting reaction products were concentrated by immunoprecipitation before the gels were loaded. The reaction mix was incubated at 37°C, the time was optimized for each experiment. The AICD production remains linear for 6 hours at saturating substrate. The reaction specificity was confirmed by running identical parallel reactions that have been saturated with inhibitors specific for γ-secretase; 10 µM LY-411,575 and 10 µM DAPT [3], [4].

### AICD detection with anti-flag αM2 monoclonal antibody or autoradiography with 125-I

AICD assays using western-blots with anti-flag αM2 monoclonal antibody, or 125-I labelled C99 autoradiography were performed as earlier described [22], [37]. To keep the C99 bands visible on gels in difference to the previous studies the reaction aliquots were not subjected to methanol /chloroform extraction. Briefly, the samples were separated on Nu-PAGE 12% Bis/Tris/MES/SDS-page gels (Invitrogen) at 150 V for 55 min. For 125-I-C99 assays the gels were dried and exposed for 1–2 hours to europium intensifying screens for autoradiography. For western-blot assays the gel was transferred to nitrocellulose membrane (protean pore size 0.1 µm), blocked by TBS 1% BSA, and stained with anti-flag αM2 monoclonal antibody (25 nM). Following the washes with TBS +0.1% Tween 20, the membranes were subjected to 25 nM GAMIR (MolecularProbes), washed, and read using fluorescence at 800 nM. In all assays, the band intensity was determined using the “ribbon-option” in ImageQuant 5.0 program. The resulting peaks and the corresponding baseline were quantified using the “peak-fit” option in MicroCal Origin 7.0 program. The AICD was quantified by comparing its signal intensity with the intensity of the corresponding C99 band (i.e. known C99 concentration). The linear range and the signal calibration were further tested using known concentrations of C99 (as shown in Fig. 3), and proportional dilutions of the reaction aliquots.

### Aβ1-40 and Aβ1-42 detection using AlphaScreen®

Aβ 1–40 and Aβ 1–42 have been measured quantitatively following previously described AlphaScreen® approach [91], with some modifications to accommodate to our experimental needs. Briefly, AlphaScreen® signal is produced by activated oxygen in a laser induced photochemical reaction when antibodies carrying acceptor-beads and donor-beads bind two epitopes that are less than 20 nM apart. In our case, the acceptor-beads are coupled to antibodies specific for the C-terminal region of analyzed Aβ 1-x peptides, while the donor beads are coupled to antibodies specific for the N-terminal (3D6). Synthetic Aβ 1-x peptides of known concentration were use to calibrate the measured AlphaScreen® signals and the corresponding linear range (usually between 0.25 to 20 nM). It is important to notice that AlphaScreen® signal measured with synthetic Aβ standards does not accurately represent Aβ products in reaction aliquots. There are two major differences: i) C99 substrate in reaction aliquots competes with Aβ products for 3D6 antibodies, which leads to a decrease in the signal intensity and a smaller linear dynamic range; ii) aggregation between Aβ products (and possibly between C99 molecules and Aβ products) can artificially increase signal intensity, especially at the low concentrations of Aβ products. In the case of aggregation, the AlphaScreen® signal is artificially enhanced since it is not only due to antibodies that bind at the C-terminal and the N-terminal region of one Aβ product, but also due to antibodies that bind to the C-terminal and the N-terminal region of different Aβ product that are brought together by aggregation.

The problem of competition between Aβ products and C99 substrate for 3D6 antibodies can be addressed by calibrating the AlphaScreen® signal in presence of fixed concentrations of C99 substrate. In Michaelis-Menten experiments concentration of C99 substrate is varied and therefore its effects on 3D6 antibody can be variable. Thus, prior to the AlphaScreen® measurements all reaction aliquots have been diluted so that the final concentration of C99 is less than 5 nM. The corresponding standard curves were prepared with less than 5 nM C99. The effects of aggregation of Aβ products on AlphaScreen® signal were more difficult to address since the aggregation between Aβ products depends on time and the solution [56]. Those can not be replicated with confidence using synthetic Aβ peptides. We found empirically that the aggregation artifacts become increasingly more present in Aβ solutions with time. These artifacts result in an unacceptable scatter of the measured signal, and there is no linear decrease in signal intensity with proportional dilutions of the reaction aliquots. The lower the enzyme activity, the more troublesome are those effects. Thus, a standard rectangular hyperbola is not observed when reaction is increasingly less saturated with its C99 substrate (usually a lag, or abrupt stepwise changes in signal intensity are observed at the low substrate concentrations). Increasingly more serious aggregation artifacts are observed in reaction aliquots that used C99 substrate that has been fast frozen and stored at −80°C for increased time periods (especially more than a week). Such measurements gave a high scatter at the low substrate concentrations despite of a high activity at the high substrate concentrations. The AlphaScreen® readouts do not follow a linear response at any dilution of the reaction aliquots. When γ-secretase assays are performed with C99 substrate immediately after the purification, the measured reaction aliquots give the highest AlphaScreen® signal, with a very low scatter, and readout that is linearly proportional to the size of the reaction aliquot. A standard rectangular hyperbola is observed when the reaction is gradually saturated with C99 substrate.

### Analysis of Aβ 1-x peptides by Urea Gels

Urea gels were used to analyze to what extent Aβ peptides longer than Aβ 1–42 represent the total Aβ. Urea gels 8M/10 T%/5% C/ SDS-PAGE were prepared, used, and processed as earlier described [22], [49], [92]. Briefly, mini-gels were prepared in three layers, running gel Tris/H_2_SO_4_ pH = 8.1 (5.8 cm), stacking gel BisTris/H_2_SO_4_ pH = 6.7, and comb gel BisTris/Bicine pH = 7.7. The continuous voltage electrophoresis was adjusted to 100 V (65–30 mA), the run time was about 1 h 35 min, until dye front was 5 mm from the bottom edge. At the end of electrophoresis the prepared gel was transferred to PVDF membranes in 90 min using Invirtogen semi-dry transfer units. Following the transfer the membrane was boiled for 5 min in PBS, and blocked with RotiBlock® (Carl Roth) according to the manufacturer instructions. The blocked membranes were exposed to 20 nM 82E1 antibody overnight, and then washed with TBS+0.1% Tween 20, three times 10 minutes. The second membrane incubation was 4 hours long in the presence of 20 nM of biotinylated goat-antimouse IgG prepared in TBS (TBS, Tris/HCl pH = 7.6, 150 mM NaCl). The third incubation was with 10 nM streptavidin-horse-radish- peroxidase. The gel was developed using a gel imaging devices with CCD camera and chemiluminescence reagents according to the manufacturer instructions. The band intensity on the acquired gel images were quantified using the “ribbon-option” in ImageQunat 5.0 program, and the resulting peaks and the corresponding baselines were resolved and quantified using the “peak-fit” option in MicroCal Origin 7.0 program.

### Preparation of Aβ 1-x standards

All Aβ 1-x standards were prepared by the solid phase synthesis as a lyophilized powder. The powder was dissolved in a small amount of trifluoro-cyclohexane, that was subsequently slowly evaporated under argon, re-suspended in TBS, and frozen on −80°C in aliquots that were used only once.

### C99 dimerization/oligomerization assays using AlphaScreen® approach

PerkinElmer's acceptor and donor beads were coupled to 3D6 antibodies following the manufacturer's instructions. The prepared acceptor and donor beads were incubated with different dilutions of fresh C99 substrate that gave high activity in different activity measurements (Fig. 3). After three hours of incubation 20 µL aliquots were used to measure the AlphaScreen® signal using 384 well plates and PerkinElmer instrument. It is important to notice that if the concentration of C99 is more than 20 fold higher than the concentration 3D6 antibody the signal will start dropping even in the case of interaction. The decrease in the signal intensity is a result of dilution of the labeled antibodies in the large excess of interacting C99 molecules.

### Data Analysis

All experimental results were analyzed using MicroCal Origin 7.0 program, using non-linear least square regression, and the equation that represent specific mechanism. All results are reported as the best fit value ± standard error with two sigma confidence intervals shown in square brackets (i.e [x, y]) [69]. Briefly, the standard error indicates precision (i.e. random errors) for each method, the two sigma confidence intervals indicate the ability of given experimental setup to resolve specific parameters. The random error for presented techniques is low, as indicated by a low scatter from the best fit values. We optimized our experiments to maximize the resolution of each parameter by increasing the number of independent data points with even distribution throughout the full range of measured profiles (i.e. maximizing the number of degrees of freedom [69]).

The relative intensity of AICD, C99 and Aβ 1-x products in different gels was quantified by transforming the individual bands into a series of peaks using the “ribbon option” in program ImmageQuant 5.0. The resulting peaks and the corresponding baselines were quantified using the “peak-fit” option in MicroCal Origin 7.0 program. The linear dynamic range for each measurement was tested by quantified by using different dilutions of the analyzed samples.

The data representing pre-steady-state burst have been analyzed using the corresponding equation [62], [63]:
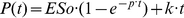
(1)


where [*P*](*t*) is product at time *t*, *ESo* is the apparent initial enzyme-substrate concentration based on the burst intercept (p. 238 in [62]), *p* is the pre-steady-state rate, and *k* is the steady-state rate (i.e. *k = kcat·ESo*, *kcat*, the turnover rate, *ESo* [62]). The initial reaction lag was analyzed using a model equation for enzyme hysteresis [63]:
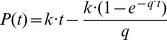
(2)


where [*P*](*t*) is product at time *t*, *k* is the catalytic rate constant in the steady-state (i.e. *k = kcat·ESo*, *kcat*, the turnover rate, *ESo* the initial concentration of enzyme-substrate complex). The lag transition rate is labeled as *q*. All standard dose response curves were analyzed using a standard equation [69]:
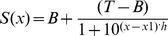
(3)


where, *x* represents logarithm of inhibitor concentration, *S(x)* is measured signal at inhibitor concentration *x*, *B* is the signal at inhibitor concentration zero, *T* is the highest signal achieved. Logarithmic values of the IC50 are labeled with *x1*, while *h* represent the corresponding Hill's coefficient. Changes in catalytic rates as a function of enzyme saturation with its C99 substrate was analyzed using nonlinear least square and the Michaelis-Menten equation [62]:
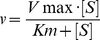
(4)


*v* is measured reaction rate, *Vmax* is the maximal rate at the saturating substrate, *Km* is the Michaelis-Menten constant, [S] is concentration of C99 substrate. Apparent dissociation constant *Kd* for interaction between C99 molecules was calculated by deriving a quadratic equation [69] that is specific for dimerization:
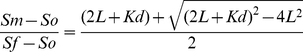
(5)


where *Sm* represent measured signal, *So* initial signal and *Sf* the final signal at the plateau. *L* represents C99 concentration and *Kd* corresponds to the apparent dissociation constant.

## Supporting Information

Figure S1 **Numerical simulation of different Aβ catalytic intermediates in γ-secretase reaction.** (A-C). Computer programs KINSIN [98] and GEPASI [99] use numerical simulation to generate model results that allow comparisons between the proposed enzymatic mechanism and the actual experimental results (Fig 1 and 2). (A) The scheme shows catalytic cycle for the processive cleavages of C99 substrate by γ-secretase in its most basic form (Fig. 10). Such cycle is easy to simulate, the enzyme (E) has only one substrate (S), and the catalytic intermediates have only two possible fates: irreversible proteolytic cleavage or irreversible dissociation (Fig. 10). The simulation of relative difference between different Aβ catalytic intermediates is based on the ratio between the cleavage rates and the dissociation rates, following the experimental data shown in supplement figure 3. For example, if Aβ 49 is 5% of the total Aβ, the ratio between the rate of cleavage (i.e. Aβ 49 to Aβ 46) and the rate of dissociation of Aβ 49, should be 95 over 5. The same approach is continued to simulate the time profiles for Aβ 46, Aβ 43, Aβ 40, and Aβ 37 using the percentages numbers shown in the scheme. The experimentally measured time profiles for AICD and Aβ 40 (Fig 1) are the reference for the required time scale, i.e. the values for the chosen rate constants are calculated so that the simulated profiles for AICD and Aβ 40 profiles maximally overlap with the experimental profiles (k1 rate corresponds to pre-steady-state rate in Table 1, the steady-state rate is the slowest step in the cycle). Finally, the extent of accumulation of each intermediate depends on ratio between its rate of formation and rate of degradation (as illustrated in detail on p. 145 in Ref. [62]). Those ratios are not known for the catalytic intermediates of γ-secretase . Thus, we chose to simulate situation with 1∶1 ratios which represents intermediate accumulation of each intermediates (i.e. the rate of formation and degradation of Aβ 49, Aβ 46, Aβ 43 are equal). The results in Fig. 2 indicate that it is very likely that the actual ratio is in favor degradation (i.e. minimal accumulation of reaction intermediates as shown on p. 145 in Ref. [62]). (B-C). Panel B shows an attempt to simulate data in Fig. 1, the panel C shows only the early data points. The simulation shows that the longer Aβ are most dominant in the early stages of the reaction and progressively decline with the reaction progress to steady-state. The actual experiments showed an opposite situation (Fig 1–2), Aβ 40 dominates in the pre-steady-state, and that longer Aβ fragments start to accumulate only with the reaction progress to the steady-state (Fig 1 and 2). Thus, γ-secretase can not be described as an enzyme that follows the same processive mechanism in the pre-steady-state and the steady state. The discrepancy between the model data and the experimental data supports our proposal that progress of γ-secretase reaction in time leads to a change in the enzyme's ability to process and hold the longer Aβ catalytic intermediates.
(DOC)

Figure S2 **Titration of γ-secretase activity using potent γ-secretase inhibitor LY-411, 575.** Highly potent enzyme inhibitors can be used to estimate concentration of active enzyme (p 206. in ref [62]). LY-411, 575 is one of the most potent γ-secretase inhibitors, its IC50 in cell-based assays is about 100 pM. Thus, LY-411,575 can be used to estimate γ-secretase concentrations when the active enzyme concentration is above 100 pM. We find that about 1 to 2 nM of LY-411,575 can completely abolish γ-secretase activity in CHAPSO enriched membranes with total protein concentration equal to 0.25 mg/ml (O) and 0.09 mg/ml (•). Thus, the highest concentration of the active enzyme in our assay can not be more than 1 to 2 nM.
(DOC)

Figure S3 **Analysis of different Aβ/total AICD ratios from the published studies** [**37**] **.** To our knowledge only one of the published studies analyzed saturation of γ-secretase with its C99 substrate by measuring Km profiles for its different products [37]. Here we show that the data from Kakuda and co-authors lead to the same conclusion as our data in Fig. 4A. The reported Km and Vmax values (shown in table) can be used to calculate the corresponding saturation curves (eqn. 4 in methods [62]), and the calculated saturation curves can be used to analyze of different Aβ/total AICD ratios. (A–B) Similar to Fig. 4A, the panels show that increase in the enzyme saturation with its C99 substrate leads to decrease in dominance of Aβ 40 product. At the lowest saturation 40% of initial AICD cleavages will result in Aβ 40 as the final cleavage product (Fig. 10), only about 2% of initial AICD cleavages will result in Aβ 48 as the final cleavage product (Fig 10). (C–D) Panels show that the decrease in Aβ 40 product predominantly correlates with the increase in Aβ 43, and Aβ 49 products. Aβ 49–46–43–40 are on the same cleavage path [37], [40], [48]–[50], thus the decrease in Aβ 40 can be attributed to the premature release of the nascent Aβ 43 and Aβ 49 catalytic intermediates (Fig. 10). To lesser degree, increase in γ-secretase saturation with it C99 substrate leads to increase in Aβ 42, Aβ 45 and Aβ 48. Aβ 48–45–42 are on a different cleavage path than Aβ 40 [37], [40], [48]–[50]). Thus, to a lesser degree, saturation with C99 substrate can affect the initial γ-secretase-C99 complex so that the initial cleavage takes place at the Aβ 48 site rater than the Aβ 49 site (Fig. 10). In sum, the data from Kakuda and co-authors [37] show that increase in the enzyme saturation with its C99 substrate leads to increase in Aβ42/Aβ 40 ratio as a result of decrease in Aβ 40 and increase in production of the longer more hydrophobic Aβ products.
(DOC)
