# Supplementary material for: Modulation of γ-Secretase Activity by Multiple Enzyme-Substrate Interactions: Implications in Pathogenesis of Alzheimer's Disease
Source: PLoS One. 2012 Mar 30;7(3):e32293. doi: 10.1371/journal.pone.0032293 (PMC3316526; doi:10.1371/journal.pone.0032293)
Supplement: Figure S1 — Numerical simulation of different Aβ catalytic intermediates in γ-secretase reaction. (A-C). Computer programs KINSIN [98] and GEPASI [99] use numerical simulation to generate model results that allow comparisons between the proposed enzymatic mechanism and the actual experimental results (Fig 1 and 2). (A) The scheme shows catalytic cycle for the processive cleavages of C99 substrate by γ-secretase in its most basic form (Fig. 10). Such cycle is easy to simulate, the enzyme (E) has only one substrate (S), and the catalytic intermediates have only two possible fates: irreversible proteolytic cleavage or irreversible dissociation (Fig. 10). The simulation of relative difference between different Aβ catalytic intermediates is based on the ratio between the cleavage rates and the dissociation rates, following the experimental data shown in supplement figure 3. For example, if Aβ 49 is 5% of the total Aβ, the ratio between the rate of cleavage (i.e. Aβ 49 to Aβ 46) and the rate of dissociation of Aβ 49, should be 95 over 5. The same approach is continued to simulate the time profiles for Aβ 46, Aβ 43, Aβ 40, and Aβ 37 using the percentages numbers shown in the scheme. The experimentally measured time profiles for AICD and Aβ 40 (Fig 1) are the reference for the required time scale, i.e. the values for the chosen rate constants are calculated so that the simulated profiles for AICD and Aβ 40 profiles maximally overlap with the experimental profiles (k1 rate corresponds to pre-steady-state rate in Table 1, the steady-state rate is the slowest step in the cycle). Finally, the extent of accumulation of each intermediate depends on ratio between its rate of formation and rate of degradation (as illustrated in detail on p. 145 in Ref. [62]). Those ratios are not known for the catalytic intermediates of γ-secretase . Thus, we chose to simulate situation with 1∶1 ratios which represents intermediate accumulation of each intermediates (i.e. the rate of formation and degradation [file pone.0032293.s001.doc]

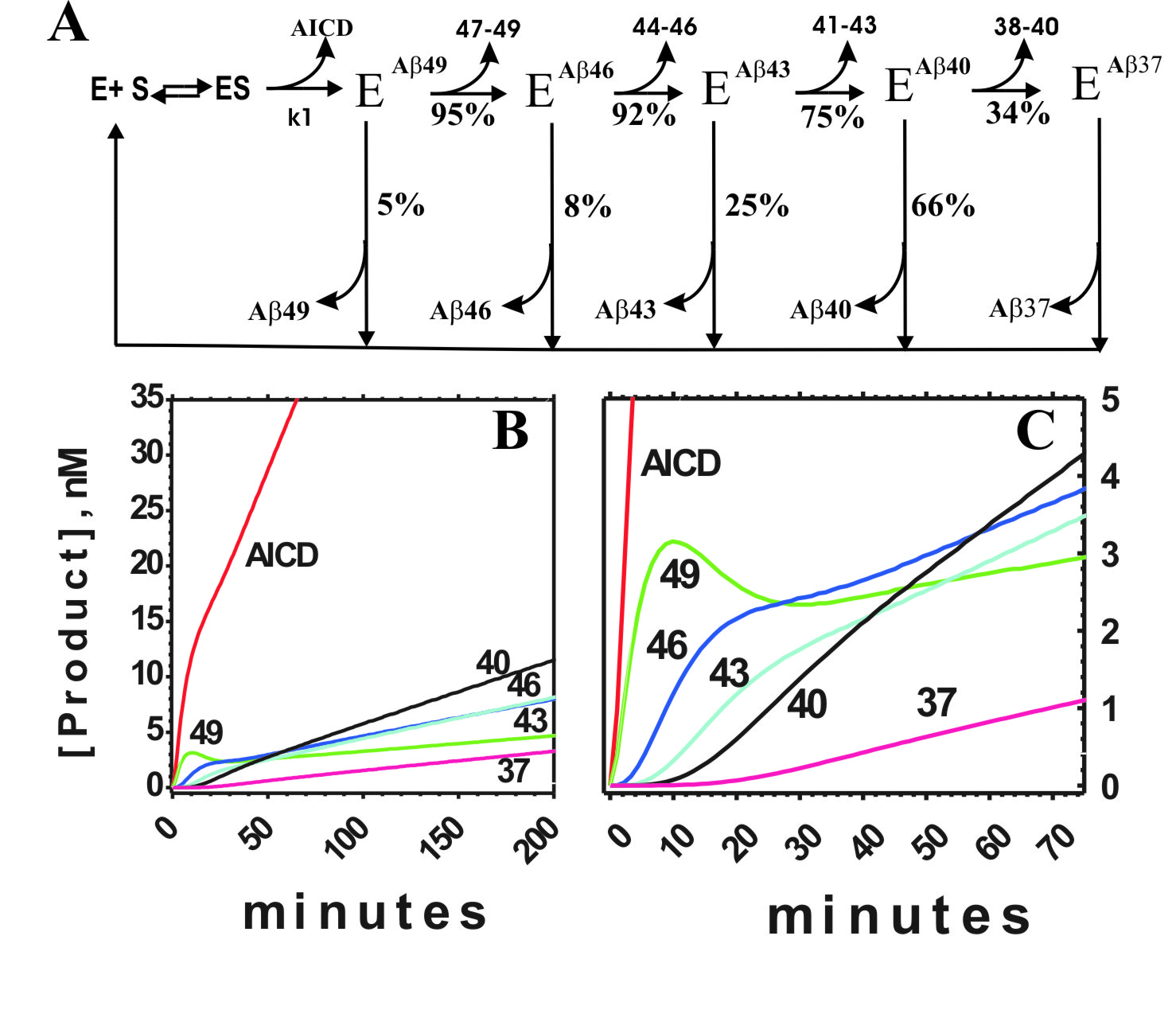


**Supplement Fig S1. Numerical simulation of different Aβ catalytic intermediates in γ-secretase reaction**.
