# Supplementary material for: Modulation of γ-Secretase Activity by Multiple Enzyme-Substrate Interactions: Implications in Pathogenesis of Alzheimer's Disease
Source: PLoS One. 2012 Mar 30;7(3):e32293. doi: 10.1371/journal.pone.0032293 (PMC3316526; doi:10.1371/journal.pone.0032293)
Supplement: Figure S2 — Titration of γ-secretase activity using potent γ-secretase inhibitor LY-411, 575. Highly potent enzyme inhibitors can be used to estimate concentration of active enzyme (p 206. in ref [62]). LY-411, 575 is one of the most potent γ-secretase inhibitors, its IC50 in cell-based assays is about 100 pM. Thus, LY-411,575 can be used to estimate γ-secretase concentrations when the active enzyme concentration is above 100 pM. We find that about 1 to 2 nM of LY-411,575 can completely abolish γ-secretase activity in CHAPSO enriched membranes with total protein concentration equal to 0.25 mg/ml (O) and 0.09 mg/ml (•). Thus, the highest concentration of the active enzyme in our assay can not be more than 1 to 2 nM. (DOC) [file pone.0032293.s002.doc]

**Supplement Fig S2.** **Titration of γ-secretase activity using potent γ-secretase inhibitor LY-411, 575.**
