# Supplementary material for: Modulation of γ-Secretase Activity by Multiple Enzyme-Substrate Interactions: Implications in Pathogenesis of Alzheimer's Disease
Source: PLoS One. 2012 Mar 30;7(3):e32293. doi: 10.1371/journal.pone.0032293 (PMC3316526; doi:10.1371/journal.pone.0032293)
Supplement: Figure S3 — Analysis of different Aβ/total AICD ratios from the published studies [37] . To our knowledge only one of the published studies analyzed saturation of γ-secretase with its C99 substrate by measuring Km profiles for its different products [37]. Here we show that the data from Kakuda and co-authors lead to the same conclusion as our data in Fig. 4A. The reported Km and Vmax values (shown in table) can be used to calculate the corresponding saturation curves (eqn. 4 in methods [62]), and the calculated saturation curves can be used to analyze of different Aβ/total AICD ratios. (A–B) Similar to Fig. 4A, the panels show that increase in the enzyme saturation with its C99 substrate leads to decrease in dominance of Aβ 40 product. At the lowest saturation 40% of initial AICD cleavages will result in Aβ 40 as the final cleavage product (Fig. 10), only about 2% of initial AICD cleavages will result in Aβ 48 as the final cleavage product (Fig 10). (C–D) Panels show that the decrease in Aβ 40 product predominantly correlates with the increase in Aβ 43, and Aβ 49 products. Aβ 49–46–43–40 are on the same cleavage path [37], [40], [48]–[50], thus the decrease in Aβ 40 can be attributed to the premature release of the nascent Aβ 43 and Aβ 49 catalytic intermediates (Fig. 10). To lesser degree, increase in γ-secretase saturation with it C99 substrate leads to increase in Aβ 42, Aβ 45 and Aβ 48. Aβ 48–45–42 are on a different cleavage path than Aβ 40 [37], [40], [48]–[50]). Thus, to a lesser degree, saturation with C99 substrate can affect the initial γ-secretase-C99 complex so that the initial cleavage takes place at the Aβ 48 site rater than the Aβ 49 site (Fig. 10). In sum, the data from Kakuda and co-authors [37] show that increase in the enzyme saturation with its C99 substrate leads to increase in Aβ42/Aβ 40 ratio as a result of decrease in Aβ 40 and increase in production of the longer more hydrophobic Aβ products. (DOC) [file pone.0032293.s003.doc]

|  | **AICD** | **Aβ40** | **Aβ42** | **Aβ43** | **Aβ45** | **Aβ48** | **Aβ49** |
| --- | --- | --- | --- | --- | --- | --- | --- |
| **Km, µM** | 508 | 408 | 609 | 725 | 788 | 754 | 933 |
| **Vmax, pmol min^-1^** | 653 | 205 | 173 | 180 | 56 | 25 | 42 |

**Supplement Figure S3**. **Analysis of different Aβ/total AICD ratios from the published studies [37]**
